# Simultaneous Assignment and Structure Determination of Proteins From Sparsely Labeled NMR Datasets

**DOI:** 10.3389/fmolb.2021.774394

**Published:** 2021-11-24

**Authors:** Arup Mondal, Alberto Perez

**Affiliations:** The Quantum Theory Project, Department of Chemistry, University of Florida, Gainesville, FL, United States

**Keywords:** molecular dynamics, protein structure determination, sparse NMR, MELD, REMD

## Abstract

Sparsely labeled NMR samples provide opportunities to study larger biomolecular assemblies than is traditionally done by NMR. This requires new computational tools that can handle the sparsity and ambiguity in the NMR datasets. The MELD (modeling employing limited data) Bayesian approach was assessed to be the best performing in predicting structures from sparsely labeled NMR data in the 13th edition of the Critical Assessment of Structure Prediction (CASP) event—and limitations of the methodology were also noted. In this report, we evaluate the nature and difficulty in modeling unassigned sparsely labeled NMR datasets and report on an improved methodological pipeline leading to higher-accuracy predictions. We benchmark our methodology against the NMR datasets provided by CASP 13.

## Introduction

NMR is one of the most widely used biophysical techniques to study macromolecules such as proteins and nucleic acids. It operates under many possible regimes, leading to dynamic, structural, and functional information over a wide range of timescales from the picosecond to the sub-millisecond timescale (Kempf and Loria, 2002). We focus here on the use of NMR for structure determination. The three-dimensional arrangement of atoms in a protein dictates its biological functionality such as catalytic activity, transportation, or mechanism of actions in diseases like cancer, Parkinson’s, or Alzheimer’s, to name a few (Chaudhuri and Paul, 2006). Understanding the behavior of proteins allows us to hypothesize about how to control their activity, how they interact with other biomolecules, or how to mutate them to manipulate their functionality, which in turn will accelerate the drug discovery process and help us develop new therapeutic methods (Senior et al., 2020). The PDB database contains ∼160,000 protein structures, being an essential resource to inquire about protein structure–function relationships. Of these structures, 90% are solved by X-ray crystallography and about 8% by NMR (Guzenko et al., 2020). Even though this is a small fraction, protein structures solved by NMR are important, as not all proteins can be crystallized. They mostly represent underrepresented folds and solvated structures that correspond to physiological conditions (Fowler et al., 2020). CryoEM is an emerging technique that is increasingly providing high-resolution structures—it typically deals with larger molecular assemblies and, in some cases, suffers from heterogeneous map resolution across the sample.(Abriata and Dal Peraro, 2020).

NMR uses chemical shift data from NOESY experiments to identify contacts between atoms that are far away along the sequence. In two-dimensional H^1^-H^1^ NOE experiments, every hydrogen atom pair within 6 Å gives a signal in the NMR spectra, and the intensity of the signal decreases proportionally with *r* ^6^, where *r* is the distance between the interacting atom pair (Bax, 1994; Clore and Gronenborn, 1998; Bax and Grzesiek, 1993). The resulting spectra become intricate as many atom pairs are found interacting in a protein (Gaalswyk et al., 2018) and give rise to peaks in the spectra. The challenge is to identify the pair of atoms that gives rise to each peak. This is done by *sequential assignment* using through-bond NMR experiments, where each peak results in multiple ambiguous interpretations. Assigning each signal and solving its ambiguity is a time-consuming step in traditional NMR structure determination, although software helps to automate the process (Huang et al., 2005; Huang et al., 2015). Given that proteins have large conformational landscapes, the presence of each contact reduces the viable conformational space and accelerates the process of exploration and identification of relevant biological states. When enough contacts are determined, they can be used to produce NMR ensembles that are representative of the native state. Historically, molecular mechanics strategies such as simulated annealing approaches have been combined with NMR data to produce restrained conformational ensembles (Clore and Gronenborn, 1998). For a typical protein solved by NMR, we need ∼20 restraints per amino acid (Aiyer et al., 2021). In this scenario, the protein force field and sampling techniques have limited contribution to the overall structure but are useful to get the right stereochemical properties and reduced numbers of steric clashes. On the opposite end, where NMR data are sparse, a good force field and sampling strategy are needed to identify plausible macromolecular structures.

Despite significant successes, structure determination by NMR is currently challenged by the size of the macromolecules. The sharpness of NMR peaks depends on the relaxation time. The faster the molecules relax, the broader the peaks become. As a system gets to a larger size, it possesses slower tumbling rates which result in short *T*_2_ (transverse magnetization) values, leading to fast relaxation of the excited states and broader peaks (Foster et al., 2007; Clore and Gronenborn, 1998). Broader peaks lead to increasing peak overlaps, limiting the identification of individual peaks. Generally, NMR of proteins beyond 200 residues is not successful due to such peak broadening. Carrying out NMR at high temperatures would circumvent the slow tumbling rate, but most proteins are not stable under such non-physiological conditions (Foster et al., 2007).

The question we address here is to expand the use of NMR beyond the current size limitation. The NMR field is already using sparsely labeled proteins (e.g., ILVA labeling) to increase the quality of the spectra on larger proteins (Tugarinov and Kay, 2003). The trade-off involves the reduction of amounts of restraints per amino acid and a non-homogeneous distribution of the information along the protein chain. Eventually, this reduced number of restraints might not be enough to build a structural model with automated structure calculation tools (Huang et al., 2005; Huang et al., 2015). This has limited the progress of NMR-driven structure determinations of larger proteins. On the flip side of the coin, macromolecules such as proteins have an inherently large number of conformations they can adopt, too many to explore them systematically and identify the native one. This has limited computational prediction of protein structures until recent machine learning–based developments (Jumper et al., 2021; Baek et al., 2021). However, these predictions are not always correct and need to be validated with real data. Thinking beyond proteins, NMR is applicable to DNA, RNA, complexes, modified proteins, and other systems which machine learning might not have been trained for yet (Becette et al., 2020). This is where integrative methods which combine experimental data and computer simulation play an important role. These methods can use limited information from experiments and combine them with a physical model to produce atomic resolution structures (Webb et al., 2018; Gaalswyk et al., 2018) by filling the blanks present in the experimental dataset. From a computational point of view, focusing on the region of the landscape compatible with NMR data greatly improves the chances of sampling native structures.

MELD uses Bayesian inference to integrate data from different experimental sources with an atomistic force field to predict structures (MacCallum et al., 2015; Perez et al., 2016). MELD simultaneously solves the ambiguity of the data and produces structures which are compatible with NMR data. While more computationally expensive than traditional methods, it leverages a physics-based model to fill the gaps in regions where no data points exist (MacCallum et al., 2015). Such datasets have been introduced in the CASP (Critical Assessment of Structure Prediction) event, where MELD was independently scored as the leading methodology to handle this type of data (Robertson et al., 2019). CASP is a worldwide protein structure prediction competition event that happens biennially (Kryshtafovych et al., 2019), where predictor groups are asked to produce structural models for sequences that they are provided with and that are being independently solved experimentally. At the end of the competition, CASP assesses predictor groups with respect to the experimental structures with different matrices. This has provided a way of assessing improvements in the field through blind, independent testing, which has led to the successes of AlphaFold, AlphaFold2, and RoseTTAFold (Senior et al., 2020; Jumper et al., 2021; Baek et al., 2021). CASP-NMR is a sub-category of CASP (CASP11 and CASP13) which provides sparse, ambiguous, and noisy unassigned NMR data along with the protein sequences and asks groups to solve the corresponding three-dimensional structures. In the 13th edition of CASP, despite the successes of this approach in the NMR category, the determined structures, in some cases, were of lower accuracy than those predicted in the absence of data (Kryshtafovych et al., 2019; Sala et al., 2019). In this work, we take the previous success and develop new strategies for better integration between data and MELD. Through this process, we test multiple new protocols to identify and overcome bottlenecks that have prevented higher accuracy predictions in the past. We have selected a protocol that systematically outperforms other approaches in our benchmark set.

## Methods

### MELD Approach for NMR Data

MELD combines semi-reliable data (i.e., sparse, ambiguous, and noisy data) from different sources (experimental, bioinformatics, or machine learning) with molecular dynamics simulations. This approach is ideally suitable for dealing with the noisy and ambiguous datasets arising from deuterated protein samples (Robertson et al., 2019), such as those provided in CASP13 (Kryshtafovych et al., 2019; Sala et al., 2019; Huang et al., 2019). MELD uses a Bayesian framework to enforce data into simulation, mathematically, as follows:

$$p\left(x|D\right)=\frac{p\left(D|x\right)p\left(x\right)}{p\left(D\right)}∼p\left(D|x\right)p\left(x\right),$$ (1)

where x represents a particular conformation at a timestep given by an atomistic force field. *D* represents corresponding NMR data. *p* (*x*) is the *prior* distribution, given by the Boltzmann distribution, based on the force field we use. *p* (*D*) is a data likelihood that can be regarded as a normalization factor. *p* (*D*|*x*) is the likelihood of the data given a structure and represents the probability of satisfying a certain subset of data (the size of the subset is provided as a parameter, see the protocol section). *p* (*x*|*D*) is the posterior distribution that we sample from; it provides the distribution of sampling certain structures given the fraction of the data that we enforce. MELD uses a Hamiltonian and temperature replica exchange protocol (H,T-REMD) (Sugita and Okamoto, 1999; Fukunishi et al., 2002) to sample the energy landscape efficiently. The temperature changes geometrically across the replica exchange ladder, while the Hamiltonian changes nonlinearly as previously described (MacCallum et al., 2015). At the highest replica index, we enforce high temperature and no restraints, favoring the exploration of the energy landscape, while at the lowest replica index, we sample the lowest temperature in the ladder and enforce the data with full restraints, leading to the exploitation of minima that are compatible with a subset of data.

We consider a hierarchy in the dataset which we call a collection of NOESY peaks. Each peak contains a group of possible pairs of atoms that could give rise to the NOESY signal, based on their chemical shift. Each group contains at least one possible interpretation, but often it has many more. Each possible contact in the group is represented as a restraint between two atoms. During the simulations, given the current structure, all restraints in each group are evaluated, and the lowest energy restraint is selected to represent the group. Then the group energies are ranked and the lowest energy groups up to the selected accuracy of the NOESY peaks are selected. These are the restraints that will be enforced until the next timestep. Each replica will have a different set of restraints active. At high replica indices, we expect the subsets of data enforced to change easily between timesteps.

### NMR Data and Datasets Used

We selected the 13 proteins from the NMR-assisted prediction category in the 13th edition of CASP. There is a wide distribution of lengths, ranging from 80 to 326 residues. CASP provided four different types of data along with sequences for each target: ambiguous H^1^-H^1^ NOE data, dihedral data generated from the TALOS program (Shen and Bax, 2015), residual dipolar couplings (RDCs), and evolutionary contacts. We only used NOE data and dihedral data for each protein target. Synthetic NOE data derived from chemical shift were provided for all targets except n1008 and N1008, where real NMR data were provided (Sala et al., 2019; Robertson et al., 2019). The data were generated by NMR experts to represent the problems of ambiguity and noise in the form of missing or spurious peaks (Sala et al., 2019) typical in these datasets. For targets n1008 and N1008, real NMR data were provided (Sala et al., 2019; Robertson et al., 2019). The provided peaks originated from backbone amides and from methyl hydrogen sidechains of isoleucine, leucine, valine, and alanine (ILVA) residues. For target n1008, we were provided the complete peak list with ambiguity. The provided dihedral data were calculated from chemical shifts using the TALOS program. All NMR data used in this work can be accessed *via* the official CASP13 webpage http://www.predictioncenter.org/casp13/index.cgi or, alternatively, by using 10.5281/zenodo.3471415 link (Sala et al., 2019).

On one hand, the provided NOE peak lists contain many possible restraints because of the ambiguity and added noise (Sala et al., 2019). On the other hand, the dataset is sparse because ILVA residues are not always homogeneously distributed throughout the protein sequence, and the correct set of restraints is not enough by itself to fully determine the native structure. The nature of the ambiguity-given chemical shifts poses a challenge for our physics-based approach, where restraints that are local along the sequence provide no reduction of the conformational space but can incorrectly bias the ensembles when they are incorrect. We thus generated four possible NOE datasets for each target using the data provided during CASP. In the first dataset, we removed all peaks which contain at least one interpretation that could be satisfied by residues closer than four residues along the sequence. For the remaining peaks, we mapped the sidechain H atoms to the corresponding heavy atoms, adding $1\overset{{}^{\circ}}{A}$ to the NOE. This reduces the ambiguity due to symmetric hydrogens (e.g., in a methyl group). We named this dataset the *4-residue ambiguous dataset*. A second dataset, named the *4-residue true dataset*, was built by MDTraj (McGibbon et al., 2015) using the native structures to identify all contacts present in the *4-residue ambiguous dataset* that can simultaneously be satisfied in the native structure. For the third dataset, named the *4-residue true clustered dataset*, we clustered contacts from the second dataset in 10 clusters with a *k*—*means* clustering protocol and chose the centroid of each cluster for each target. Considering the larger size of targets N1005 and N0981D3, we chose 10, 25, 50, and 100 clusters (or restraints) for N1005 and 10, 20, 30, and 50 clusters (or restraints) for N0981D3. The fourth dataset was built as the first dataset, by removing only peaks that could be interpreted with contacts along the same residue. We call it the *1-residue ambiguous dataset*.

### MELD Protocols

We use the MELD (MacCallum et al., 2015) plugin to the OpenMM molecular dynamics package (Eastman et al., 2017) to carry out H,T-REMD simulations with the ff14SB (Maier et al., 2015) (for amino acid side chains) and ff99SB (Hornak et al., 2006) (for backbones) AMBER force field and the GBNeck2 implicit solvent model (Nguyen et al., 2013; Onufriev, 2008). Each protein was simulated with all four NMR datasets mentioned above. We started the simulations from the extended chain as produced by *tleap* (Case et al., 2005). With the first and second NMR datasets, we designed nine different protocols combining three different temperature ranges (300–550, 350–550, and 400–550 K) and three different force constants (87, 350, and 700 *kJmol* ^−1^ *nm* ^−2^) along the replica ladder. Under these simulation setups, violating an NMR peak by $1\overset{{}^{\circ}}{A}$ will add an energy penalty of 0.435, 1.75, and 3.5 *kJmol* ^−1^ for the 87, 350, and 700 *kJmol* ^−1^ *nm* ^−2^ protocols, respectively, which is lower than 1 *k*_*B*_ *T* for protocols with the first two force constants. For the third and fourth datasets, we chose the best performing protocol on the previous datasets which corresponds to the temperature range 400–550 K and the force constant of 350 *kJmol* ^−1^ *nm* ^−2^.

We tested a total of 20 protocols for each protein. All simulations have 30 replicas where the lowest replica index corresponds to the lowest temperature and is then geometrically increased to the maximum of the range at the 12th replica—and is maintained at the highest temperature for higher replica indexes. Force constants are scaled nonlinearly using a scalar that ranges from 0 at the highest replica to 1 at the 12th replica—it is maintained at 1 at replicas below the 12th replica. As mentioned earlier, at the higher replica indexes, the high temperature and non-enforcement of NMR data allow broad sampling of the conformational space, and at lower replica indexes, MELD starts identifying the best interpretation of the data compatible with the physics model. As temperature is reduced, the structures are refined.

For the first and fourth NMR datasets, we enforced an accuracy of 70 and 80% of total peaks, respectively, and considered that each peak had to be represented by at least one restraint from the ambiguous list. For N1005, we trusted 70% for both datasets as trusting 80% resulted in failed simulation due to large forces. For the two 1,008 targets, we trusted only 20 and 50%, respectively—as our initial analysis showed noise in these real datasets (Table 1). For the second and third NMR datasets, we enforced all the restraints as they were pre-filtered to be correct in the native structure. An exception was made for N0981D3, where we enforced 80% due to large forces at higher accuracy values resulting in simulations failing. The backbone dihedral predictions from TALOS were trusted at 80% confidence and predicted secondary structures from PSIPRED (McGuffin et al., 2000) with 60% confidence.

**TABLE 1:** Noise in NMR datasets and simulations.

	—	4-residue ambiguous			1-residue ambiguous		
Target	Simulation	Total	True	Trusted	Total	True	Trusted
—	Length (*μs*)	Peaks	Peaks	In MELD	Peaks	Peaks	In MELD
n1008	0.5	840	29%	20%	1887	53%	50%
N1008	0.5	205	27%	20%	394	58%	50%
N0968s1	0.5	219	77.2%	70%	597	87%	80%
N0968s2	0.5	219	77.4%	70%	460	87%	80%
N0957s1	0.5	325	91%	70%	944	97%	80%
N0980s1	0.5	207	67%	70%	457	83%	80%
N0981D1	0.5	147	75.6%	70%	259	85%	80%
N0981D2	0.5	177	76.6%	70%	272	82%	80%
N0981D3	1.0	553	74.7%	70%	974	82%	80%
N0981D4	0.5	220	73.3%	70%	454	85%	80%
N0981D5	0.5	227	78.4%	70%	543	86%	80%
N0989	0.5	505	69%	70%	1,146	82%	80%
N1005	1.5	1,200	66.3%	70%	3,100	72%	70%

The timestep was set to 4.5 *f*s by hydrogen mass repartitioning (Hopkins et al., 2015). Simulation lengths are summarized in Table 1, where each simulation ran for at least 0.5 *μ*s, and some were extended to improve convergence depending on the type of data and system size.

### Stability Simulations

We performed implicit solvent simulations starting from the native structure of each protein to test the stability with the physical model we used using the AMBER MD package (Case et al., 2005). We used two different protocols for the stability test: with NMR data (*4-residue true dataset*) and without NMR data. In both cases, we perform 100-ns AMBER simulation with the GBneck2 implicit solvent model and the ff14SB amber force field at 300 K. We report the backbone root mean square deviation (RMSD) from native along the trajectory in the results section.

### Clustering

At the end of each MELD simulation, we performed hierarchical agglomerative clustering on the second half of the five lowest temperature replicas using CPPTRAJ (Roe and Cheatham, 2013). We have used two different clustering protocols for the aforementioned four datasets. Both protocols use average linkage for calculating distances between clusters with a distance cutoff of $1.5\overset{{}^{\circ}}{A}$ for simulations with no ambiguity (second and third NMR datasets) and $2\overset{{}^{\circ}}{A}$ for simulations with ambiguous data (first and fourth NMR datasets) considering the fact that ensembles generated with ambiguous data are broader than those with true data. We report the centroid of the most populated cluster as our prediction (*top*1), or the best centroid from the top five population clusters (*top*5).

### Refinement

We refined our *top*1 predicted structures from the inexplicit solvent using the OPC water model (Izadi et al., 2014) and the ff19SB force field (Tian et al., 2020). We used the NMR assignments that were compatible with the *top*1 cluster. We ran these simulations for 1 *μ*s at 298 K for each prediction, using hydrogen mass repartitioning and a timestep of 4 *fs*. At the end of the simulation, we performed hierarchical clustering and again selected the centroid of the top cluster as a representative structure of the ensemble.

## Results

### Sparsely Labeled NMR Leads to Heterogeneity of Data Distribution and the Ability to Determine Protein Structures

**FIGURE 1:**
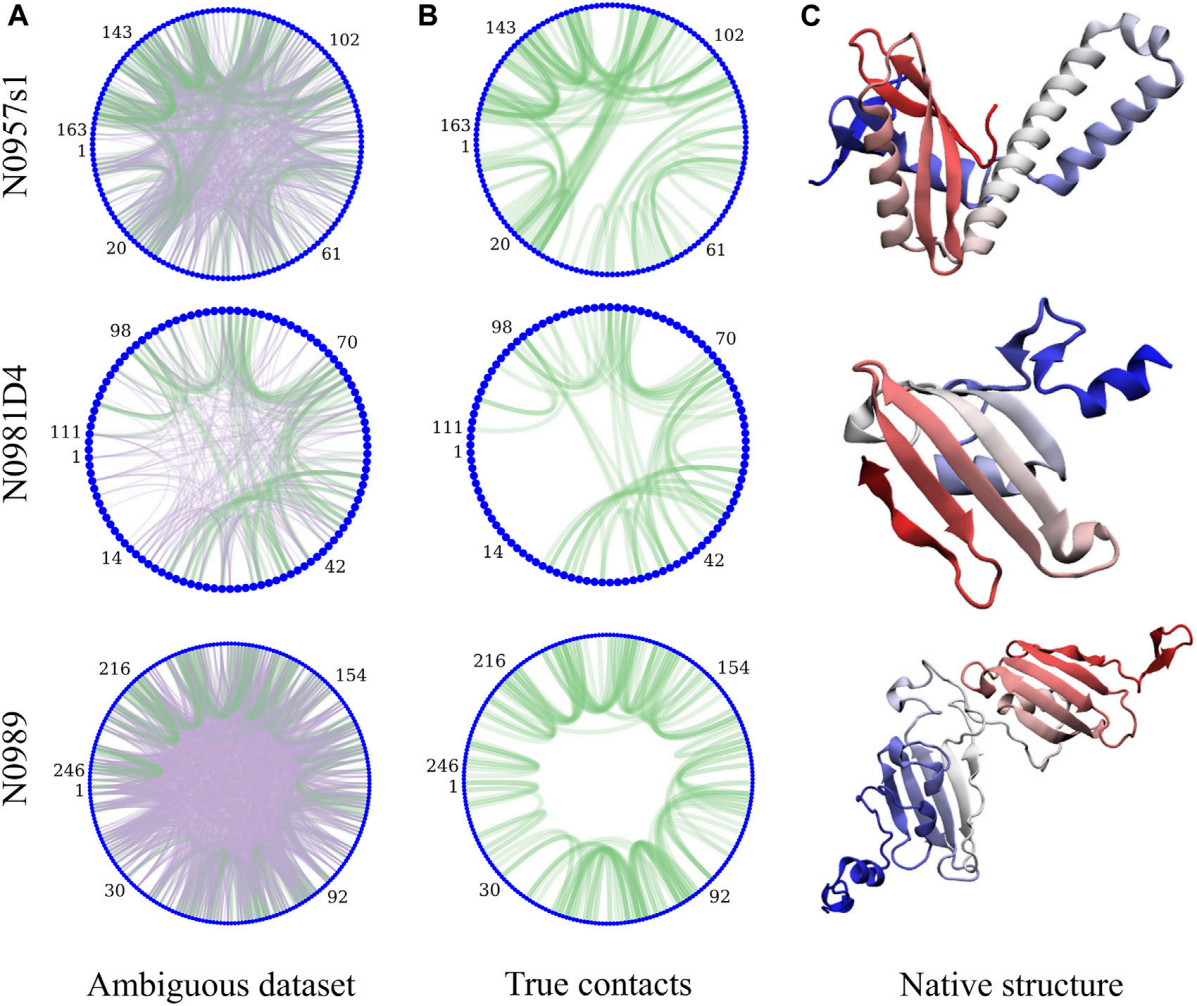
Protein topology determines the heterogeneity in the NMR datasets and their information content. The circle plots are a graph representation of the protein sequence, with each residue being a blue node. Lines represent contacts present in the dataset due to ambiguity **(A)**. Purple lines represent incorrect assignments of the peaks and green are the correct assignments. **(B)** shows only the correct assignments for clarity. **(C)** shows the experimental structures (N-termini in blue and C-termini in red).

Sparsely labeled proteins produce NMR datasets that are heterogeneous in nature due to the distribution of non-deuterated groups in residues along the sequence (isoleucine, leucine, and valine in this case). The number of peaks (Table 1) and their specific arrangement lead to a different level of ambiguity, noise, and information content. Figure 1 exemplifies the diversity in the amount of ambiguity and noise and information content in three proteins in our dataset. When the information is local or large regions of the protein have no information to guide the structure, the physics model is responsible for accurately guiding the conformational search. For protein N0957s1, the true data are distributed along the whole sequence with long- and short-range contacts present, favoring the determination of the two domains—but no contacts between the two domains limits the ability to determine their overall disposition. For target N0981D4 (Figure 1B), the ambiguity level is low and easier to solve, but the N-termini domain contains no NMR information, again resulting in a reduced ability to predict accurate conformations. Finally, the case of N0989 presents a case of high ambiguity, where the information is very localized, with few global contacts, resulting in a challenging dataset to determine the relative orientation of the domains. Details of ambiguous, true, and satisfied data are shown in Supplementary Figures S1, S13.

### Some Proteins Are Unstable With the Physical Model We Use

There were 13 datasets provided by CASP (Sala et al., 2019), corresponding to 12 different proteins. Some datasets corresponded to multidomain proteins, for which assessment was carried out for the whole assembly and for the individual domains. For the case of N0981, the protein was divided into five stable structural domains, with NMR data provided independently for each of the five domains. Our first analysis identified the correct NMR data on each dataset based on the published native structure for each protein. We then simulated each protein in the presence/absence of the true NMR data using implicit solvent MD (see methods). Supplementary Figure S4 shows that for most proteins, the force field is able to stabilize the native structure, the only exceptions being N0957, N0989, and N0981D2. As expected, simulations combining the physics model and correct assignments of NMR data produced lower RMSD distributions for all cases. However, target N0989 remained unstable with our force field combination. This sets the baseline expectation: a successful approach should be able to identify the native state in all targets, except N0989.

### Enforcing Data Into the Simulation Increases the Frustration in the Energy Landscape

By introducing data as restraints, regions of the energy landscape not compatible with data are shifted to higher energies. This produces a highly frustrated energy landscape that is harder to sample. By using a combination of temperature and Hamiltonian changes in a replica exchange approach (H,T-REMD), we can facilitate the exploration of the landscape to visit different minima (compatible with different interpretations of the NMR data). A critical point in REMD is to favor exchanges and round trips along the replica ladder to favor sampling. In this work, we explore how the energy penalty and temperature range affect the ability to identify native structures.

**FIGURE 2:**
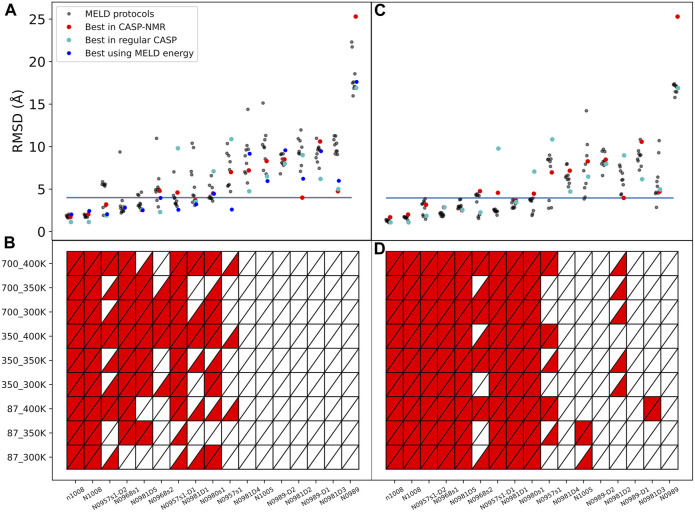
Quality of structure predictions. **(A,B)** uses the *4-residue ambiguous dataset* and **(C,D)** use the correct assignment of the NMR data (*4-residue true dataset*). We compare nine different HT-REMD protocols (black dots). The blue dots represent the lowest restraint energy structure of the best performing protocol (*k* = 350 *kJmol* ^−1^ *nm* ^−2^ and T = 400 K). The lower panels quantify each MELD protocol’s ability to identify the native structures as either red (success) or white (failure) in the *top*1 (upper triangle) or *top*5 (lower triangle) cluster centroids. The y-axis notation is k_T, where k refers to the force constant used (*kJmol* ^−1^ *nm* ^−2^) and T to the temperature in K.

The complete ensemble at the lowest replica tells us that for 10 systems, we are always able to sample native states using all protocols and using ambiguous or assigned data, whereas for target N0989, we can never sample native-like conformations. However, sampling native-like structures does not always lead to identifying them as the most populated cluster—or even amongst the top five predictions. Supplementary Figures S2, S15 show the ability to sample and identify native-like conformations in the ensembles, respectively. When we use correct assignments of the data, the preferred conformations from clustering (*top1*) are often native like, and only a few cases require looking at the *top5* clusters to identify the native structure. However, when the assignments are not given, the challenge of identifying correct restraints at the same time as the method samples correct conformations leads to a large difference between *top1* and *top5* predictions, with *top1* missing the native conformation in many cases, indicating that longer simulations would likely be needed. The disparity between the ability to sample and the ability to identify native-like conformations led us to further analysis in search for the causes. Comparing across protocols, using a higher temperature (400 K) leads to better sampling and identification of native states. An orthogonal approach is to change the force constants affecting how strongly we enforce the data. Lower restraint energies reduce the frustration but also the guiding power of the restraints. Indeed, we observe no benefit from reducing the force constants (87 kJ.mol^−1^.nm^−2^) when the data are ambiguous. Similarly, increasing the force constant (700 kJ.mol^−1^.nm^−2^) increases the frustration and backtracking issues (Capraro et al., 2008). Our preferred protocol for using unassigned NMR data uses 400 K as the lowest temperature and 350 kJ.mol^−1^.nm^−2^ as the maximum force constant in the H,T-REMD ladder. This protocol emerges as significantly better than the rest in capturing the native state in the *top*1 cluster (Figure 2). Throughout the manuscript, we report the performance of the different protocols and focus on this protocol when providing overall agreement with experimental structures.

The failure to identify native structures in all cases can in part be explained by the large restraint energies identified by our protocols, which quantify violations of the NMR peaks (Supplementary Figure S16). We find that restraint energies are a good indicator of the accuracy of the structure: low restraint energy structures from the ensemble are easily identified and often result in improved agreement with the native structure. Additionally, NMR violations can be evaluated as a post-analysis on the ensemble at different levels of accuracy. We often find that structures that are more native-like are more robust to analyzing the ensembles with high level of restraints—meaning that many contacts are synergistically satisfied, thanks to the force field, despite not being enforced in the simulation. In fact, analyzing the lowest restraint energies in this way allows us to identify structures that are more native like for several of the targets than using cluster centroids (Figure 2).

**FIGURE 3:**
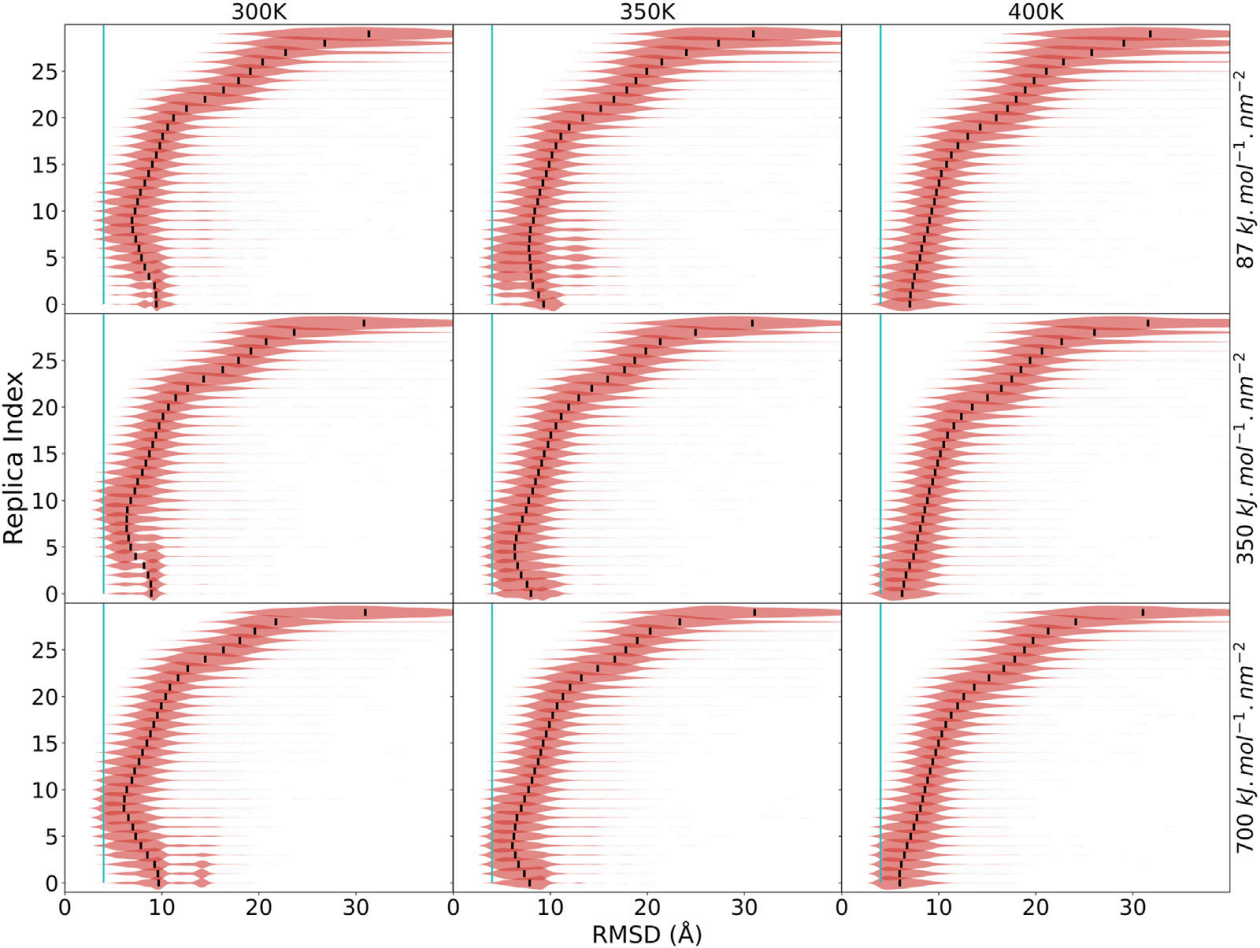
Funneling plots for target N0957s1. Each panel is a different protocol. Each line is a violin plot of the RMSD distribution at that replica, with the median of the distribution marked in black. The vertical cyan line shows RMSD = 4 Å.

However, we also see instances of restraints that cannot be satisfied because they would require chains to cross each other, causing backtracking issues (Capraro et al., 2008). The amount of data is thus not the only indicator of our ability to capture the native state, as greater amounts of data can lead to greater bottlenecks and backtracking issues. We rationalize that better protocols will present a more smooth funneling behavior, leading from unfolded states to the native state. We thus quantify the funneling of each system by following the RMSD distribution of the protein across replicas. At high replica indices, we see broad RMSD distributions, which narrow down as the replica index is reduced. The median of the distribution at each replica should also be closer to the experimental structure as the replica index decreases. We indeed find very different performances of the protocols under this metric (Figure 3). A second approach to reduce the frustration is to reduce the number of restraints used in simulations. We find that the information content of a set of restraints is very dependent on the distribution of the restraints along the chain. Thus, some restraints are redundant and help little in reducing the conformational space. Clustering the correct assignments of the NMR data significantly reduces the frustration in the system and allows for more efficient simulations. However, the ensembles are larger and the results are not as close to the native structure (Supplementary Figure S17)—these types of simulations would need longer simulation times to converge. However, the quality of the structures provides a good starting point for refinement using the whole NMR dataset.

### Multi-Domain Proteins

When analyzing the funneling plots, we notice that several multimeric and multidomain proteins are sampling native-like structures in the middle replicas but not at the bottom replicas (Figure 3). Since the bottom replicas are used for clustering, we often do not make good predictions in these systems. This is especially apparent in the case of N0957s1, consisting of two domains. Only protocols with a higher temperature range have a funneling behavior. When looking at the behavior of each domain in the trajectory, we notice that domain-2 has a strong funneling behavior in all cases (Supplementary Figure 22), especially at low temperature, whereas domain-1 exhibits a pattern of falling into an alternative conformation after sampling the native one (Supplementary Figure S19). Looking at the two domains, we have a coil–coil domain packing against and alpha + beta domain through a flexible hinge. At lower temperatures and with the force field combination we are using, the method favors compact conformations, whereas at higher temperatures, it favors a more open conformation. This hinge motion is expected and can in fact be observed by looking at the first deformation direction using an anisotropic network model (Eyal et al., 2015). The experimental structure in this case is solved with a protein in between two domains present in this protein, fixing their relative position (Supplementary Figure S18). This type of behavior can also be observed in targets N1005 (Supplementary Figure S23), N0981D2 (Supplementary Figure S21), and, to a lesser extent, N0968s1 (Supplementary Figure S20) and N0981D4 (Supplementary Figure S24).

We found that explicit solvent refinement using the *top*1 structure with the assigned NMR data that MELD produces is not useful when the initial model has low accuracy (RMSD higher than $6\overset{{}^{\circ}}{A}$ ). Similarly, when the predicted structure is already very good (RMSD below $2\overset{{}^{\circ}}{A}$ ), our refinement approach is not useful. However, when the RMSD of the predicted structure is in between $2\overset{{}^{\circ}}{A}$ and $6\overset{{}^{\circ}}{A}$ , we find that the refined models have lower RMSD (at least by $0.5\overset{{}^{\circ}}{A}$ ) than the actual MELD prediction (Supplementary Figure S25).

## Discussion

Recent advances in machine learning are providing protein models at an unprecedented level of accuracy (Jumper et al., 2021; Baek et al., 2021). However, these methods do not work for all proteins, they might not get correct structural details throughout the whole sequence, and it is not always clear what conformation is generated (e.g., holo/apo in the case of complexes). Sparsely labeled NMR data bridges two worlds: on one side it allows the use of NMR for larger molecular systems, and on the other, it provides experimental information with which to validate models coming from machine learning approaches (or other methods). The data used in this study come from assuming perdeuterated protein samples, with hydrogens along the backbone amide and some methyl groups (Sala et al., 2019). The data used are not fully assigned, but rather, from the experimental NOESY peak-list and known chemical shift; each peak is assigned a set of multiple interpretations (pairs of atoms) that could give rise to the peak. Hence, the dataset is ambiguous and sparse. It is sparse because there is not enough data to completely determine the molecular system using standard NMR tools, as whole regions of the protein might not have any NOESY peak to determine the conformation. The data are additionally noisy, meaning that some NMR peaks might be erroneous and some possible interpretations in the ambiguous data might be missing from the dataset (including the correct interpretation) (Sala et al., 2019). Moving forward, models from machine learning could be helpful in reducing the ambiguity and a good starting point to solve the ensembles that agree with the sparse set of data. In this work, we focus on solving the ambiguity in the dataset and the structure starting from extended datasets. This can be helpful for situations for which no good models exist or other polymeric material for which machine learning cannot be trained due to unavailability of large databases.

Computational NMR tools such as AutoStructure, ADSP, NOAH, ARIA, or CANDID attempt to solve the ambiguity in the data in order to use them with a structure generation tool such as XPLOR/CNS or DYANA. (Huang et al., 2005, 2015; Mumenthaler and Braun, 1995; Nilges, 1995; Herrmann et al., 2002; Kuszewski et al., 2004; Brünger et al., 1998; Güntert et al., 1997). These tools require a substantial understanding of NMR data to produce a subset of possible restraints that determines the structure amongst all the possibilities defined by the ambiguity. Exploring all possible interpretations of the data is not viable, but the right subset should be compatible with the internal geometric features of a protein. We use an alternative approach, MELD, which identifies the best interpretations of the NMR data through Bayesian inferences using a physics model as a prior. Such an approach simultaneously solves the ambiguity in the dataset and the best structural ensembles compatible with different subsets of the data and the physics model. Other tools like Rosetta have been previously used to predict structures given this type of ambiguous data (Raman et al., 2010; Kuenze and Meiler, 2019). We showed during the 13th CASP event that MELDxMD significantly improved the accuracy of the produced structures over other methods. The approach was not always successful, and in some cases, the performance was below that of methods that did not use NMR data (Robertson et al., 2019).

Standard NMR approaches are not suitable for these sparse datasets. Our choice of a physics-based approach is for multiple reasons: 1) it provides a way to model regions where no data are available and 2) it provides a way to sample the ambiguity and noise in the NMR dataset. From the physics point of view, using NMR data in simulation greatly reduces the conformational landscape, but at the same time, it increases the frustration in the energy landscape, leading to backtracking issues where satisfying an NMR restraint early on might preclude satisfying others (Capraro et al., 2008). The stability test starting from the native structure is a sanity check for the procedure. We do not expect our methodology to work if the native structure is not stable under the simulation conditions. These failures can be due to the force field or the limitations in modeling the system such as using the monomer or single-domain structure instead of the whole protein assembly. This is typically a caveat in blind tests, where it is hard to tell *a priori* if the structure of the monomer will need other subunits to be stable. Indeed, in the dataset provided, T0980 (pdbid: 6GNX) is a heterotetramer, T0968 (pdbid: 6CP9) is a heterodimer (each monomer models as s1 and s2), and T0957 is a heterodimer (pdbid: 6CP8) (Supplementary Figure S18). Only T1005 (pdbid: 6Q64) and T1008 (pdbid: 6MSP) are monomers. For T0981 and T0989 complexes, there is no experimental structure published yet, but the first consists of several interacting domains, which are simulated independently. T0989 is a homotrimer, and each subunit is made of two different domains. The data provided in CASP were generated based on taking individual monomers. Our stability tests show that the presence of NMR data indeed leads to stabilizing some structures that would otherwise not be stable with the force field alone (N0957s1 and N0981D2), while for one protein (N0989), the combination of true data and the physics model we use is not enough to stabilize the native fold (Supplementary Figure S14). This failure for N0989 is not surprising as the contacts between monomers is needed to stabilize the native (homotrimer) structure. As the trimer structure has not been released to the PDB yet, we can only know the monomer structure based on the CASP-released monomeric structure.

The intensity of an NOE peak is proportional to the ensemble average distance between pairs of atoms as $<1/r^{6}>$ . This is a well-known issue for modeling NMR data (Bonvin et al., 1994), which implies that it is not always possible to satisfy all data simultaneously in a single structure (e.g., imposing restraints). When the NMR peak-assignments are known, ensemble refinement is possible and commonly used (Konrat, 2014; Ángyán et al., 2010; Crehuet et al., 2019). However, when the assignments are not known, and we sample conformational ensembles at the same time as different interpretations of the data, such ensemble averaging becomes more challenging. Our choice for computational efficiency in MELD simulations is to evaluate data for the instantaneous structure being sampled. To reduce the problems that would arise from having to satisfy all data simultaneously, we enforce a smaller subset of the data than the known accuracy in the dataset—in this way, not all correct peaks need to be simultaneously enforced. At the end of the simulations, MELD provides the most likely structures and the assignment of the NMR data. Thus, a possible last step of refinement would be able to produce an NMR ensemble from the MELD-assigned data in explicit solvent. However, the emphasis and novelty in this work are the step of producing the most accurate initial structural model and assignment of the data. Once the data are assigned, standard simulation tools that are already well tested can be used (Bonomi et al., 2016).

We emphasize the importance of curating the datasets prior to running MELD or other approaches. Mathematically, given the list of chemical shifts, it is possible that a certain peak would be satisfied by residues that are close in the sequence space—given the ambiguous list for each peak, this can be a correct interpretation or incorrect one. In our physics-based model, residues close in a sequence are statistically more likely to be in contact with each other; hence, they will be selected as low-energy restraints very often. At best, such information is not helping with the sampling, and at worst, we are selecting incorrect information that can prevent us from sampling native conformations. We thus remove all peaks where any interpretation is satisfied by atoms belonging to residues closer than four amino acids in the sequence space. We indeed find that this systematically improved predictions over the majority of the protein systems (Supplementary Figure S17). In this process, we are effectively disregarding potentially useful information, and indeed, we see a drop in the remaining peak accuracy from an initial near 90% accuracy to ∼75% accuracy (Table 1).

**FIGURE 4:**
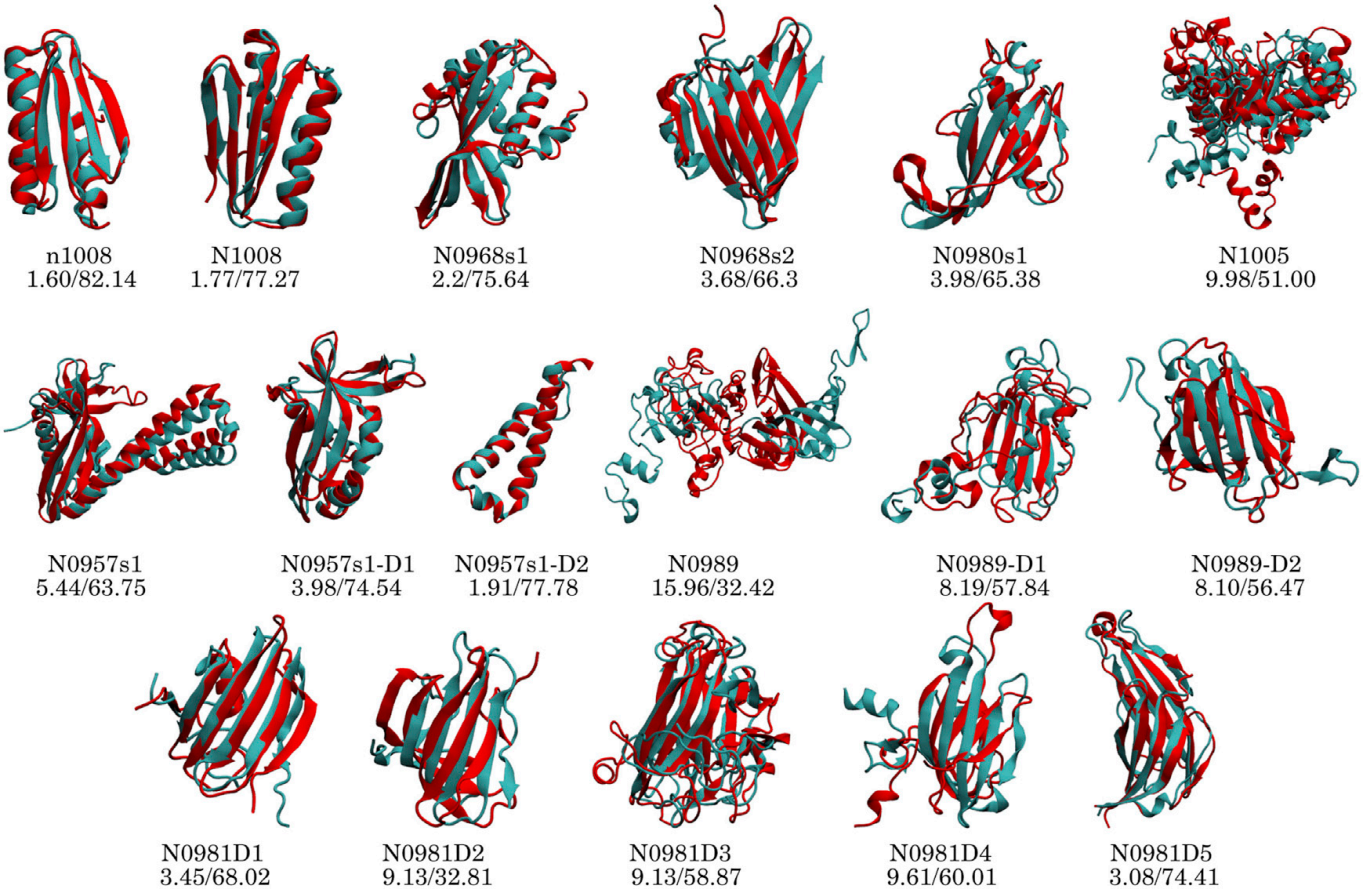
Performance of the *top*1 cluster (red) superposed on the native structure (cyan) for each protein and domain. The numbers below the target name represent the C*α* RMSD (Å) and GDT-TS values.

To identify if failures in obtaining the correct structure are due to the assignment or the sparsity, we added a new dataset in which we fed MELD the correct interpretation of the data (*4-residue true dataset*). We find that in these conditions and under an array of different MELD protocols, the results are significantly better for many proteins (Figure 2). This naturally sets the maximum expected efficiency of the approach. This dataset helps us distinguish sampling failures due to the sparsity in the dataset from those originating from the ambiguity in the data. We expect target N0989 and its two domains to be incorrect, since they are only stabilized in the trimer structure. The other two failures are N0981D2 and N0981D4. These are again difficult to evaluate in the context of the single domain. In particular, N0981D4 contains a short helical terminus that is not packed against the rest of the domain; removing this piece in the analysis results in a successful prediction of the reminder of the domain (Figure 4 and Supplementary Figure S26). MELD is thus efficient at using the sparsity in the data but is not as efficient in navigating the whole set of ambiguity in the dataset.

**FIGURE 5:**
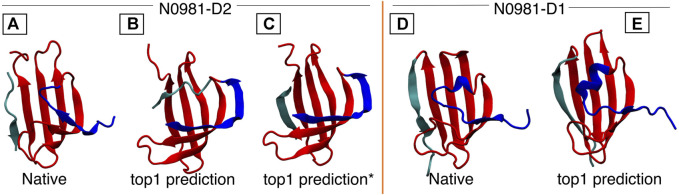
N0981D1 and N0981D2 domains are structurally similar, differing in the N-termini conformation (shown in blue). MELD captures the difference in N-termini conformation, but initial predictions fail for the cyan termini. In N0981D2, this corresponds to an independent fragment, which our standard protocol fails to bind into the main fragment. **(A)** Native structure for N0981D2. **(B)** MELD prediction for N0981D2. **(C)** MELD prediction from a modified protocol to use two fragments. **(D)** N0981D1 native structure. **(E)** MELD prediction for N0981D1.

Target N0981D2 contains two non-consecutive fragments forming a protein domain: the first is 71 residues long (residue 1–71) and the second is nine residues long (residue 72–80), where the short fragment forms a beta strand packing against the larger one. The MELD prediction has an RMSD of $9.13\overset{{}^{\circ}}{A}$ from native. Sampling failures in this case respond to how the data are distributed in the dataset and how we define the ambiguity and noise levels. First, we find that given the accuracy of the data, MELD satisfies all data on the larger fragment, with no restraints guiding sampling for the smaller one (despite correct data being available). Without enforcing the inter-fragment restraints, the small fragment could not attain its native conformation. Second, residues 1 to 14 in the first fragment form a strand, but it cannot adopt the twisted conformation found in the native structure (showed in blue in Figure 5). Few contacts in the NMR data correspond to this region (Supplementary Figure S8), further reducing the chances of exploring the native conformation. Target N0981D2 has a similar fold to that of N0981D1, and interestingly, MELD is able to capture the native form for N0981D1 (RMSD = $3.45\overset{{}^{\circ}}{A}$ ) as there are enough contacts in the range of residues 1 to 14 to stabilize the native conformation (Supplementary Figures S4, S7). We realized *a posteriori* that a modified protocol where we separately enforce inter- and intra-fragment NOESY information would have led to better results. Under this implementation, MELD models residues 72 to 80 (shown in cyan) better, and the C*α*-RMSD excluding residues 1 to 14 is now 3.6 $\overset{{}^{\circ}}{A}$ (Figure 5). This is further supported by the simulation with the *4-residue true dataset*, where we still cannot model residues 1–14 consistently, but the small fragment (residues 72–80) can be modeled better due to trusting 100% of the true contacts. However, this is not a generalizable or transferable strategy, as, in general, we only know the overall accuracy of the dataset, and we cannot say anything about the accuracy of subsets of the data *a priori.*

When the correct assignments are known, most protocols are efficient at sampling native structures. However, their performance drops significantly when it comes to identifying native-like structures on the basis of the lowest free energy structures (*top*1 or *top*5). Increasing the ambiguity reduces the sampling ability and the identification of native structures and shows more marked differences between computational protocols. Within the MELD Bayesian inference approach, there is a trade-off between the reduction of the conformational space, thanks to using restraints and the increased frustration of the system. This leads to new bottlenecks, where satisfying some restraints might limit the ability to form other restraints. Ideally, in the REMD approach, restraints that are unfavorable will be lost and new ones established. However, as walkers go up the replica ladder, all restraints loosen up and can therefore be lost. There is no mechanistic information in MELD that follows a kinetic folding pathway, but rather, the exclusion of conformation regions that are not in agreement with any subset of data. In this sense, the information is local, affecting each peak independently of the rest. We expect the physics model to account for the cooperativity of restraints. Looking at the different MELD protocols we designed, some are more suitable for sampling native states than others. Most protocols are able to sample native-like conformations in the ensembles—pointing to an efficient search strategy. But, the performance drops significantly when trying to identify the native state through clustering (even if we knew the correct assignment of the data). Interestingly, in the presence of ambiguity and noise, the best performing protocols shift to those using larger force constants and temperature. Part of it can be explained through a more funneled behavior and a greater discriminatory power from the restraints. In essence, we are shifting the balance between the restraints and the force field. In the case of traditional NMR, with 20 restraints per amino acid, the system is determined with relatively simple sampling methods and even with simple force fields. As the data become more sparse, the balance shifts more toward the force field. While some protein systems are robust independently of the method, others change significantly. This in turn ties into the distribution of the data. Some systems like N1005 or N0989 have a very local distribution of restraints, whereas others such as N1008 have many high contact-order restraints, which reduce the conformational landscape significantly. Thus, despite only 20% of the data being enforced for N1008, the results are significantly better than for other proteins of similar size.

The NMR datasets provide information for atomic contacts that are close in space but tell us nothing about long distances between residues. This, for example, translates in packing between helices, positions of loops, or relative positions between domains to have little information. On the other hand, it is very informative for packing *β*-strands. For most of the targets, the data were synthetically created based on well-determined protocols by NMR experts (Sala et al., 2019). They were derived based on the monomer structure, whereas, in reality, some of these proteins are multimeric. Considering homo-mers such as the N0989 trimer would increase the ambiguity in the dataset, where each restraint could now be satisfied in either the monomer (intra) or between monomers (inter). MELD should be able to solve this type of ambiguity as well but is likely to require longer simulations. It will be interesting to see the prevalence of such datasets in future blind events.

With the current physics-based approach, it is more important to identify the right data than to have a lot of it. For instance, clustering the correct data and using 10 contacts is already able to predict native-like topologies for many systems—with a few more clustered contacts needed for the larger proteins. One would be tempted to reduce the overall number of satisfied restraints from the larger dataset to reduce the frustration in the energy landscape, but this often leads to redundant contacts being selected. Furthermore, for the current examples, we used the known assignments before clustering the data. A maximum entropy approach that could deal with the ambiguity of the data for clustering would increase the chances of creating funneled, low-frustration energy landscapes. The information is promising for future biophysical experiments, which might produce even greater degrees of sparsity.

We focused on the optimization of sparse NOE data in this work as a source of distance restraints on the system, despite the availability of residual dipolar coupling (RDC) data for the targets we explored. RDCs inform the system of orientation preferences between residues (Lipsitz and Tjandra, 2004) and can be a useful tool to complement distance restraints in structure determination, especially for multimeric domain proteins and protein complexes. While NOE informs about residues at short distances from each other, RDCs provide structural information independently of the distance between residues (Prestegard et al., 2005). Despite their potential, they have been more challenging to incorporate into structure determination pipelines due to their dynamical and ensemble nature, with only 124 protein structures in the PDB using RDCs (Cole et al., 2021). Recent approaches for structure determination using RDCs (Cole et al., 2021; Gaalswyk et al., 2021) are promising additions to the current pipeline, synergistically working toward protein structure determination.

## Conclusion

NMR is a versatile experimental technique capable of providing structural and dynamical information. Sparse labeling techniques can provide opportunities to overcome size limitations in protein structure determination. The development of computational tools that synergize state-of-the-art computational sampling approaches with experimental data provide knowledge beyond what each independent method can accomplish. We have shown here a marked improvement over previous approaches at the problem of structure determination in the presence of ambiguous, sparse, and noisy NMR data. We believe that efforts to unify and make experimental data more accessible to non-experts like the NEF initiative will aid in the development of more computational tools working synergistically with NMR data.

## Data Availability

The datasets presented in this study can be found in online repositories. The names of the repository/repositories and accession number(s) can be found below: https://www.predictioncenter.org/casp13/targetlist.cgi?view=others&assis_type=N. The NMR data is also accessible through Zenodo: https://doi.org/10.5281/zenodo.3471415.
