# Supplementary material for: Simultaneous Assignment and Structure Determination of Proteins From Sparsely Labeled NMR Datasets
Source: Front Mol Biosci. 2021 Nov 24;8:774394. doi: 10.3389/fmolb.2021.774394 (PMC8667806; doi:10.3389/fmolb.2021.774394)
Supplement: Supplementary file 1 [file Datasheet1.pdf]

Supplementary Material

1 SUPPLEMENTARY FIGURES

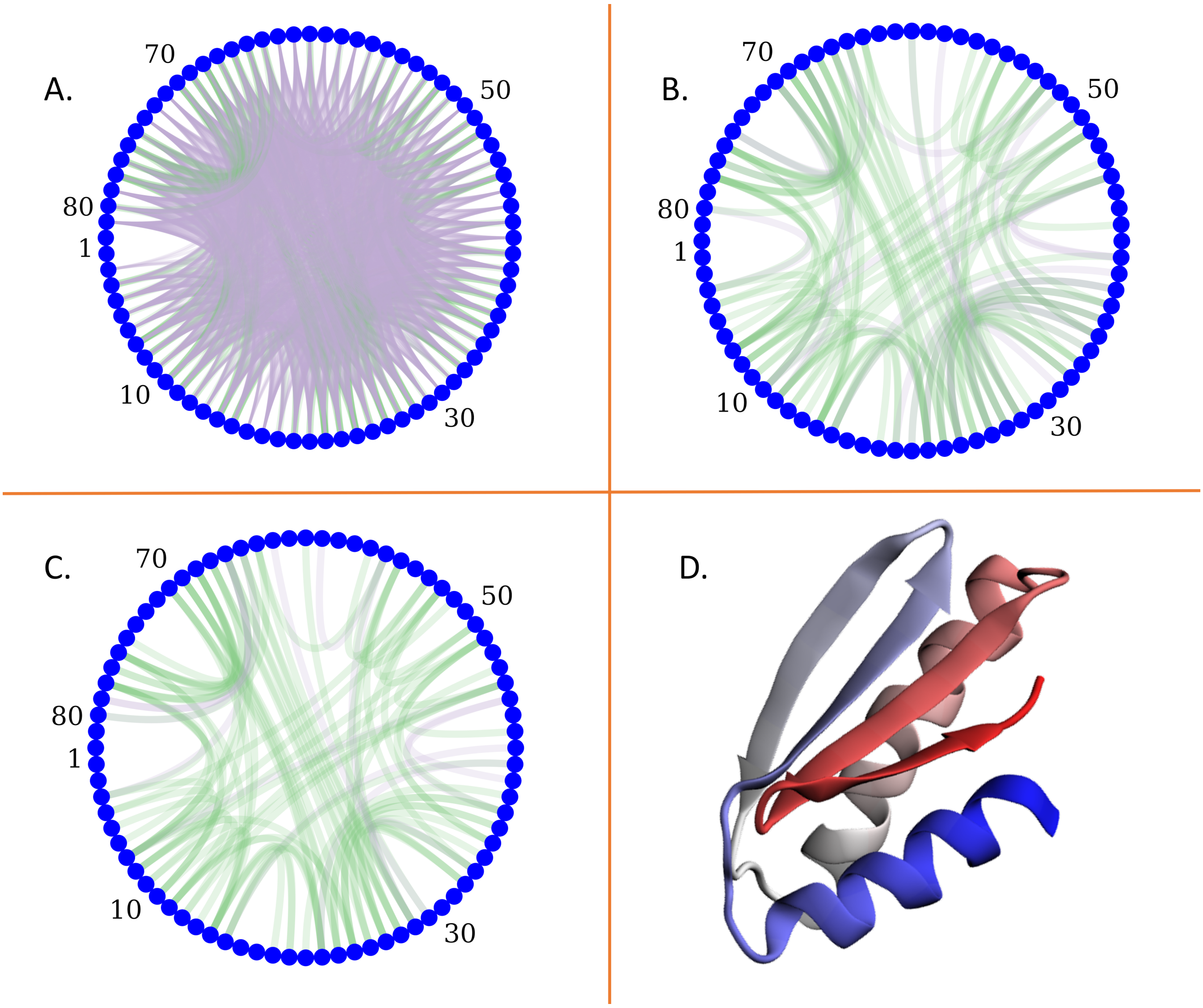

Figure S1. Target: n1008. Panels A-C show a graph representation of the protein sequence, with each residue as a blue node. Lines between residues represent contacts. Panel A: all possible contacts in the *4-residue ambiguous dataset*. Green lines represent the correct assignment of the data. Panel B: each line is a true contact in the native structure, shown as either satisfied in the $top1$ cluster (green, true positive) or non-satisfied (purple, false negative). Panel C: shows the satisfied contacts the $top1$ MELD prediction. Green contacts are present in the native structure (true positives) and purple ones are absent (false positive). Panel D: native structure using a blue (N-termini)- white - red (C-termini) coloring scheme.

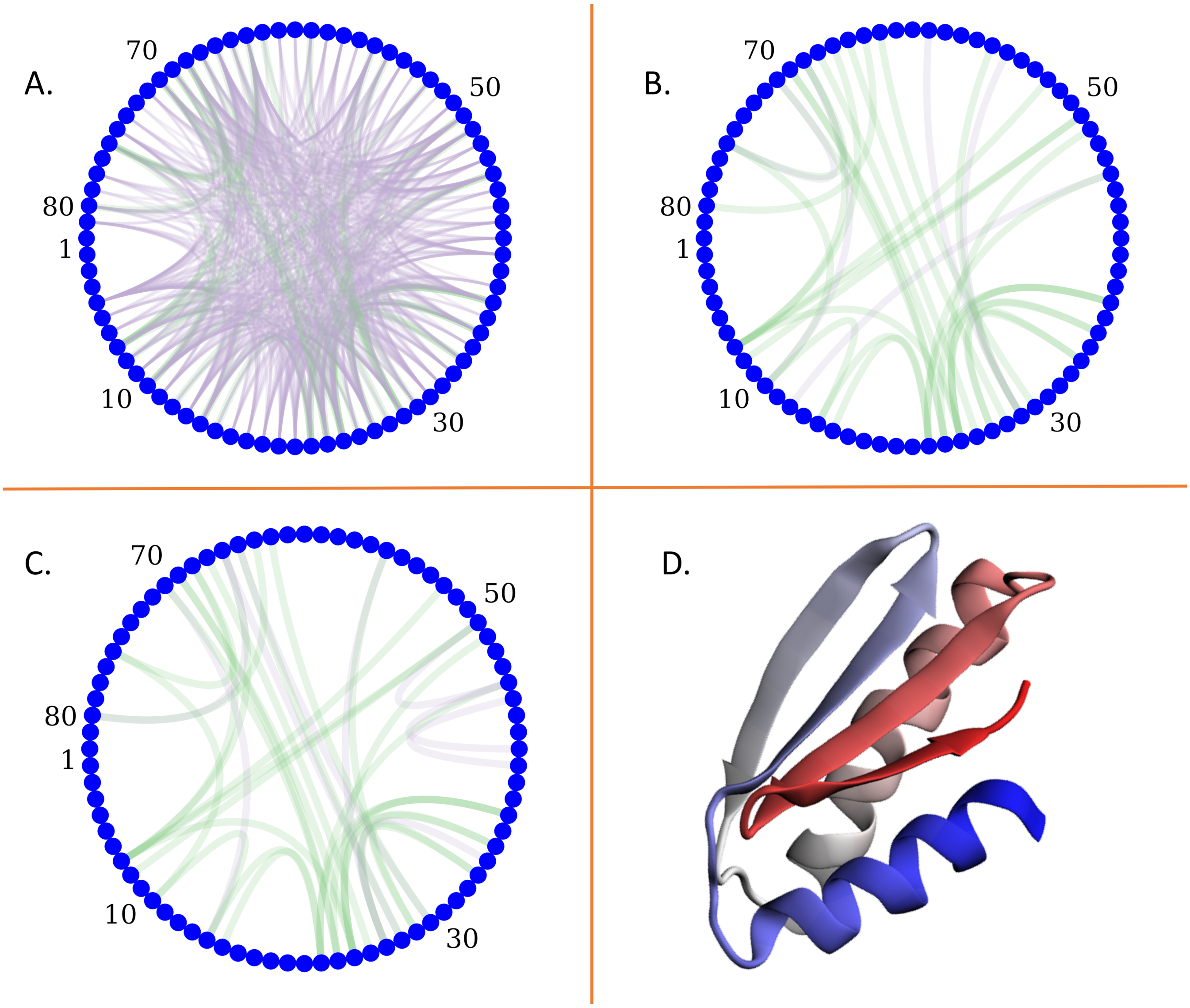

Figure S2. Target: N1008. Panels A-C show a graph representation of the protein sequence, with each residue as a blue node. Lines between residues represent contacts. Panel A: all possible contacts in the *4-residue ambiguous dataset*. Green lines represent the correct assignment of the data. Panel B: each line is a true contact in the native structure, shown as either satisfied in the $top1$ cluster (green, true positive) or non-satisfied (purple, false negative). Panel C: shows the satisfied contacts the $top1$ MELD prediction. Green contacts are present in the native structure (true positives) and purple ones are absent (false positive). Panel D: native structure using a blue (N-termini)- white - red (C-termini) coloring scheme.

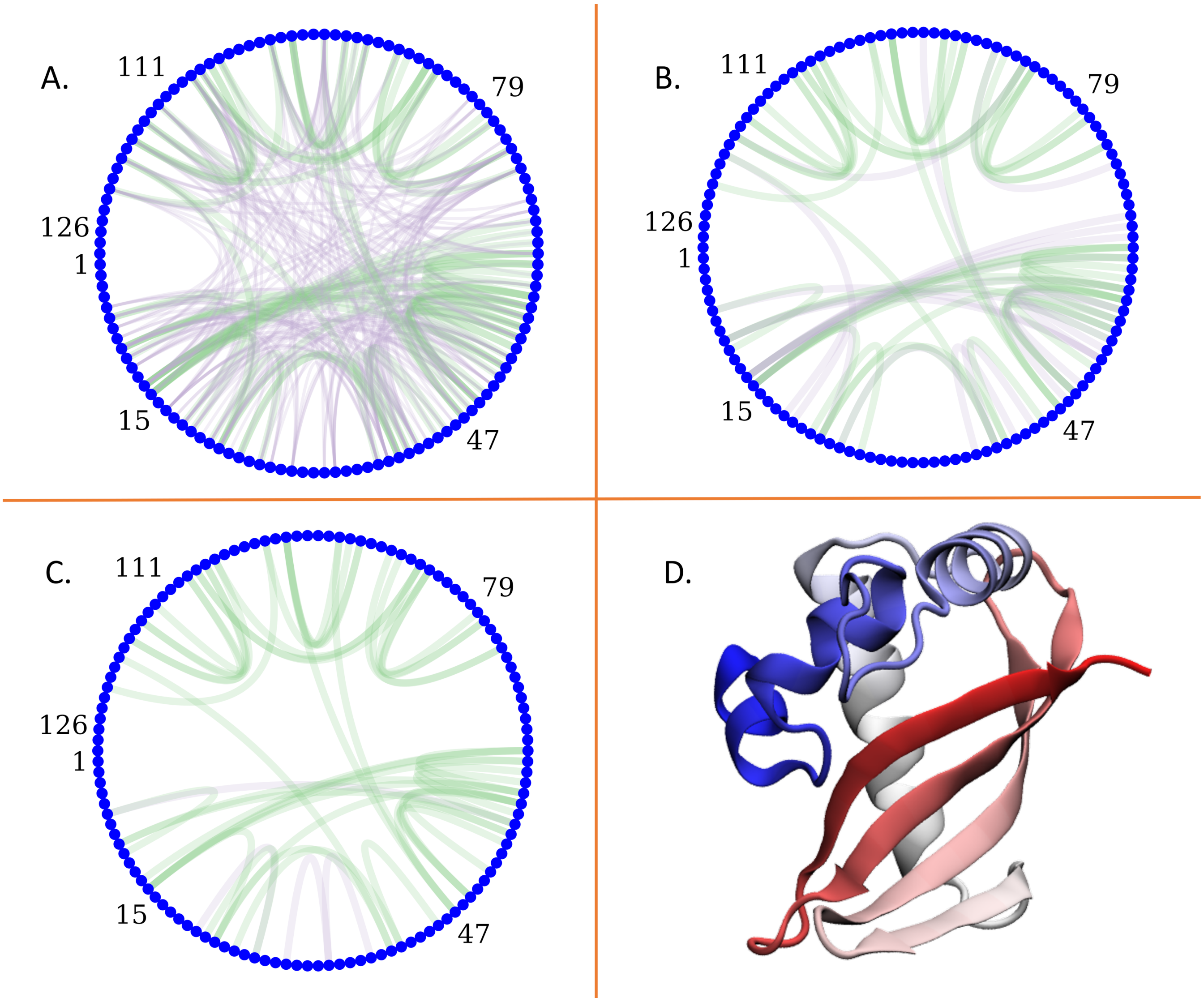

Figure S3. Target: N0968s1. Panels A-C show a graph representation of the protein sequence, with each residue as a blue node. Lines between residues represent contacts. Panel A: all possible contacts in the *4-residue ambiguous dataset*. Green lines represent the correct assignment of the data. Panel B: each line is a true contact in the native structure, shown as either satisfied in the $top1$ cluster (green, true positive) or non-satisfied (purple, false negative). Panel C: shows the satisfied contacts the $top1$ MELD prediction. Green contacts are present in the native structure (true positives) and purple ones are absent (false positive). Panel D: native structure using a blue (N-termini)- white - red (C-termini) coloring scheme.

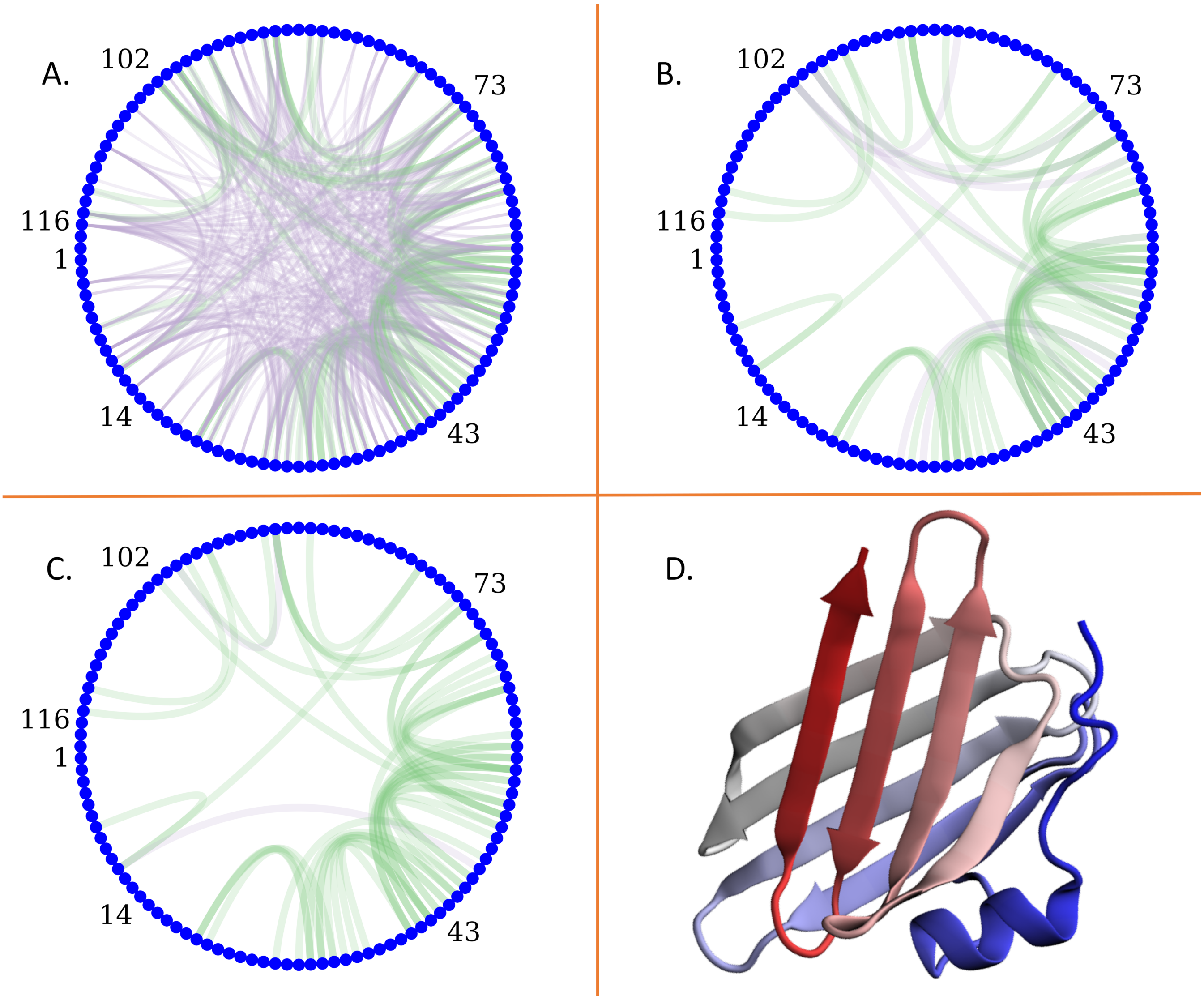

Figure S4. Target: N0968s2. Panels A-C show a graph representation of the protein sequence, with each residue as a blue node. Lines between residues represent contacts. Panel A: all possible contacts in the *4-residue ambiguous dataset*. Green lines represent the correct assignment of the data. Panel B: each line is a true contact in the native structure, shown as either satisfied in the $top1$ cluster (green, true positive) or non-satisfied (purple, false negative). Panel C: shows the satisfied contacts the $top1$ MELD prediction. Green contacts are present in the native structure (true positives) and purple ones are absent (false positive). Panel D: native structure using a blue (N-termini)- white - red (C-termini) coloring scheme.

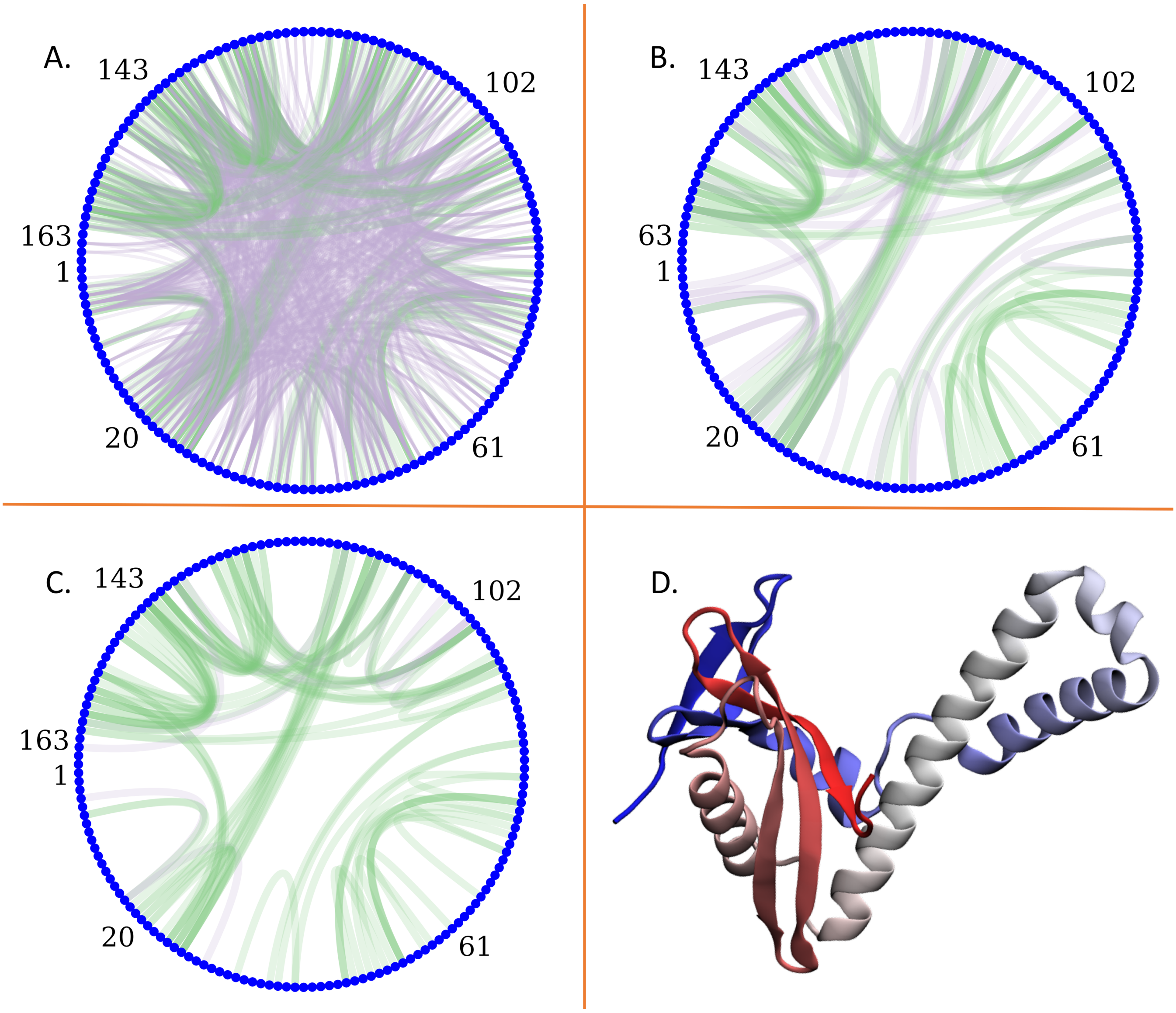

Figure S5. Target: N0957s1. Panels A-C show a graph representation of the protein sequence, with each residue as a blue node. Lines between residues represent contacts. Panel A: all possible contacts in the *4-residue ambiguous dataset*. Green lines represent the correct assignment of the data. Panel B: each line is a true contact in the native structure, shown as either satisfied in the $top1$ cluster (green, true positive) or non-satisfied (purple, false negative). Panel C: shows the satisfied contacts the $top1$ MELD prediction. Green contacts are present in the native structure (true positives) and purple ones are absent (false positive). Panel D: native structure using a blue (N-termini)- white - red (C-termini) coloring scheme.

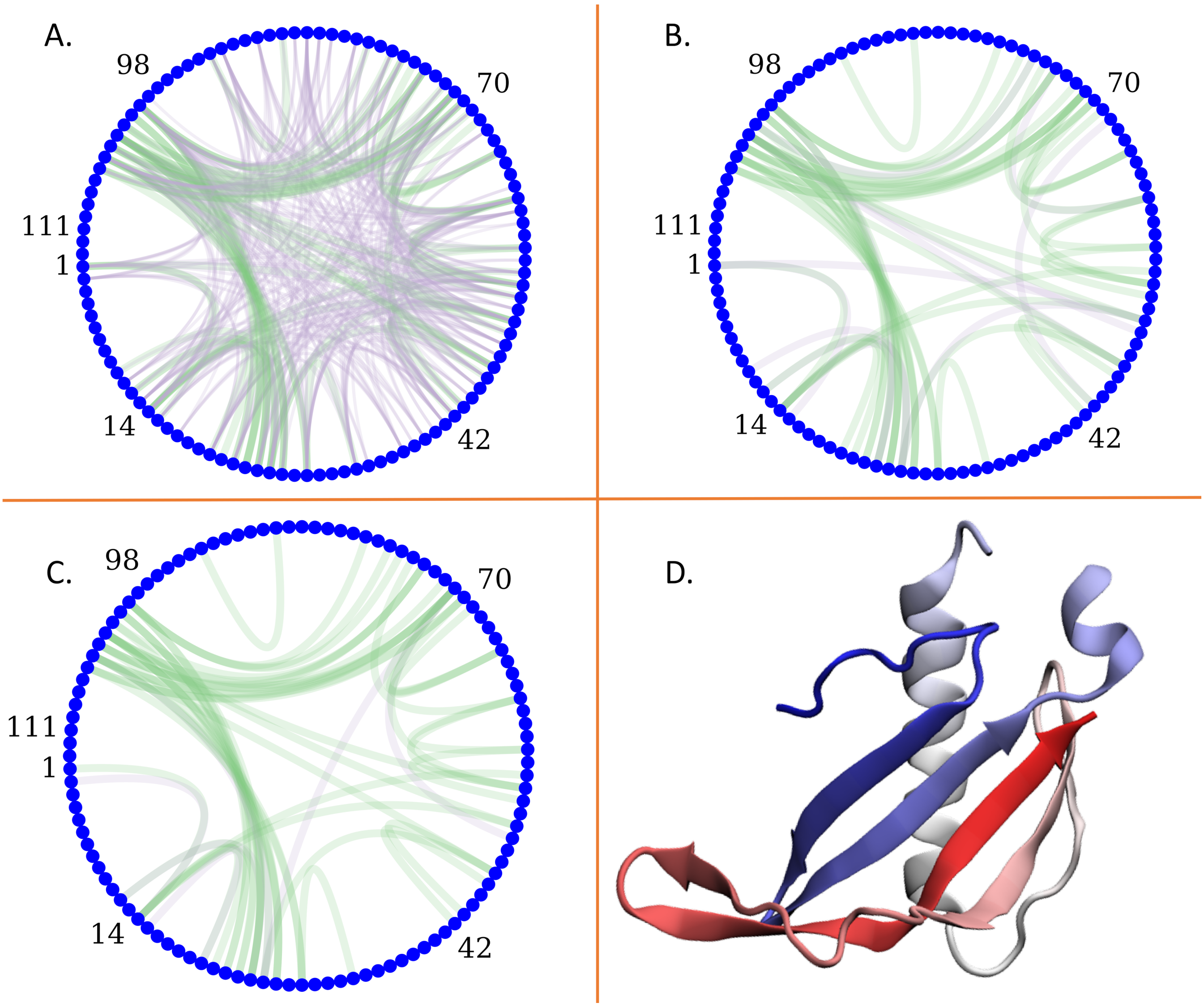

Figure S6. Target: N0980s1. Panels A-C show a graph representation of the protein sequence, with each residue as a blue node. Lines between residues represent contacts. Panel A: all possible contacts in the *4-residue ambiguous dataset*. Green lines represent the correct assignment of the data. Panel B: each line is a true contact in the native structure, shown as either satisfied in the $top1$ cluster (green, true positive) or non-satisfied (purple, false negative). Panel C: shows the satisfied contacts the $top1$ MELD prediction. Green contacts are present in the native structure (true positives) and purple ones are absent (false positive). Panel D: native structure using a blue (N-termini)- white - red (C-termini) coloring scheme.

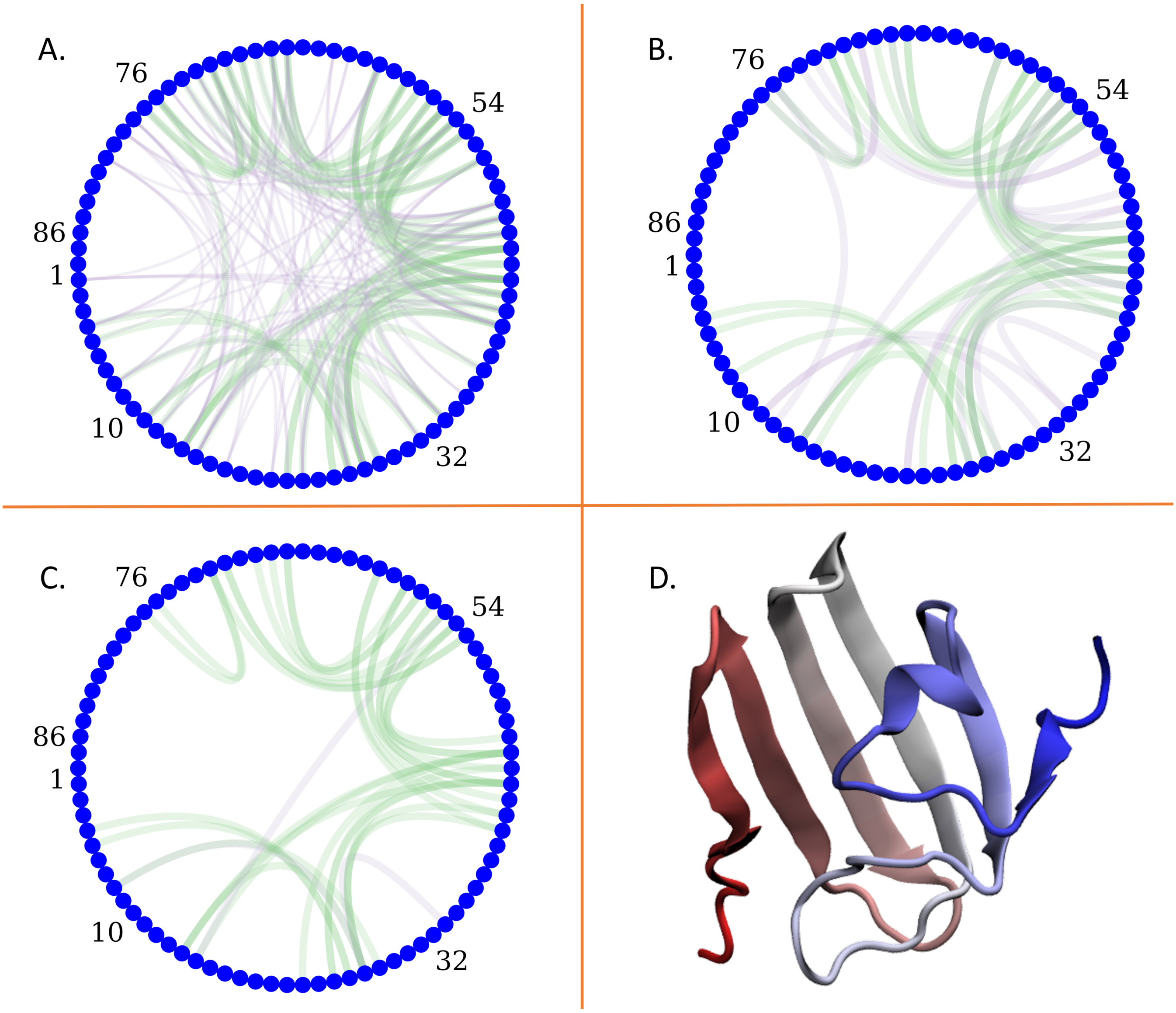

Figure S7. Target: N0981D1. Panels A-C show a graph representation of the protein sequence, with each residue as a blue node. Lines between residues represent contacts. Panel A: all possible contacts in the *4-residue ambiguous dataset*. Green lines represent the correct assignment of the data. Panel B: each line is a true contact in the native structure, shown as either satisfied in the $top1$ cluster (green, true positive) or non-satisfied (purple, false negative). Panel C: shows the satisfied contacts the $top1$ MELD prediction. Green contacts are present in the native structure (true positives) and purple ones are absent (false positive). Panel D: native structure using a blue (N-termini)- white - red (C-termini) coloring scheme.

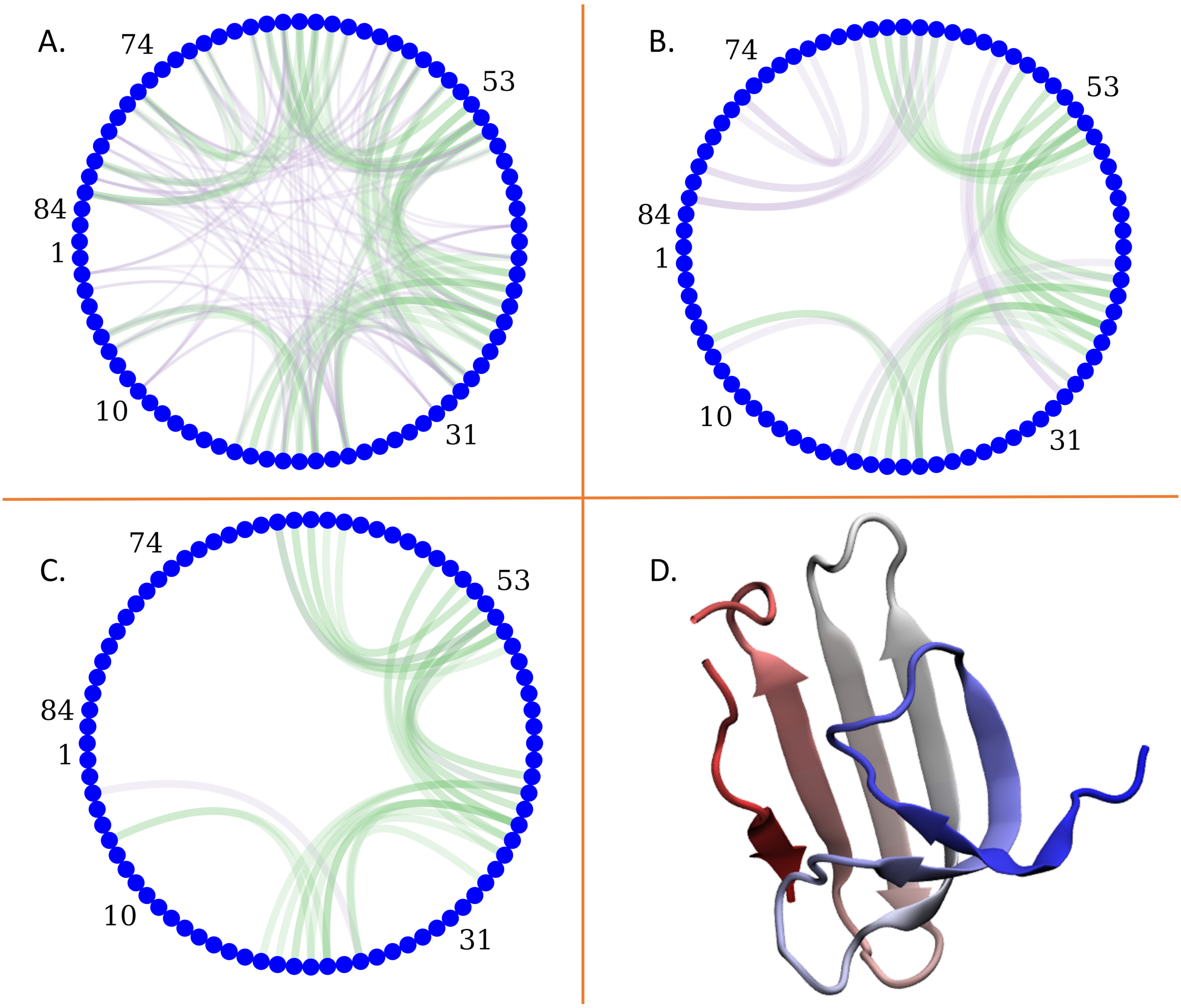

Figure S8. Target: N0981D2. Panels A-C show a graph representation of the protein sequence, with each residue as a blue node. Lines between residues represent contacts. Panel A: all possible contacts in the *4-residue ambiguous dataset*. Green lines represent the correct assignment of the data. Panel B: each line is a true contact in the native structure, shown as either satisfied in the $top1$ cluster (green, true positive) or non-satisfied (purple, false negative). Panel C: shows the satisfied contacts the $top1$ MELD prediction. Green contacts are present in the native structure (true positives) and purple ones are absent (false positive). Panel D: native structure using a blue (N-termini)- white - red (C-termini) coloring scheme.

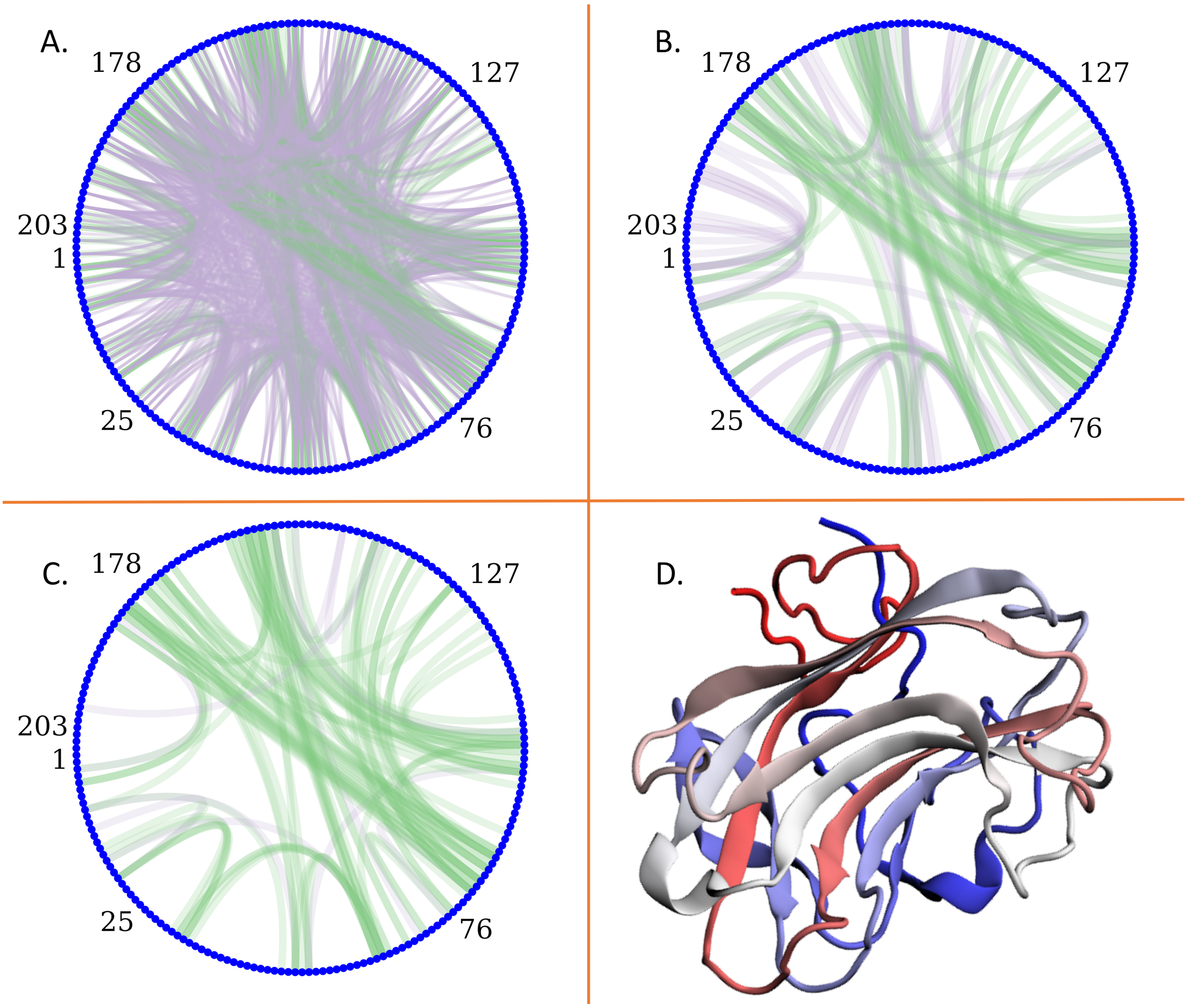

Figure S9. Target: N0981D3. Panels A-C show a graph representation of the protein sequence, with each residue as a blue node. Lines between residues represent contacts. Panel A: all possible contacts in the *4-residue ambiguous dataset*. Green lines represent the correct assignment of the data. Panel B: each line is a true contact in the native structure, shown as either satisfied in the $top1$ cluster (green, true positive) or non-satisfied (purple, false negative). Panel C: shows the satisfied contacts the $top1$ MELD prediction. Green contacts are present in the native structure (true positives) and purple ones are absent (false positive). Panel D: native structure using a blue (N-termini)- white - red (C-termini) coloring scheme.

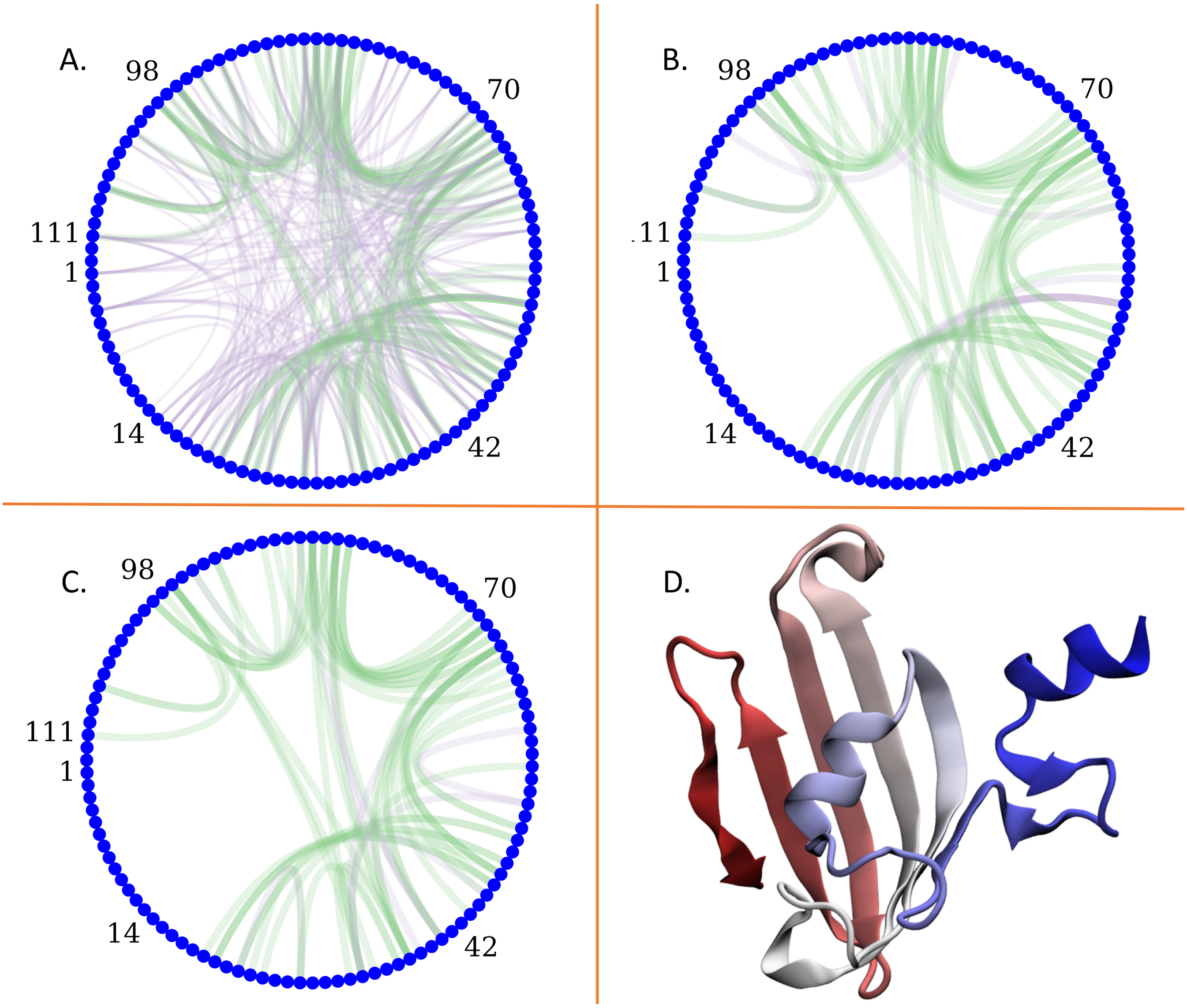

Figure S10. Target: N0981D4. Panels A-C show a graph representation of the protein sequence, with each residue as a blue node. Lines between residues represent contacts. Panel A: all possible contacts in the *4-residue ambiguous dataset*. Green lines represent the correct assignment of the data. Panel B: each line is a true contact in the native structure, shown as either satisfied in the $top1$ cluster (green, true positive) or non-satisfied (purple, false negative). Panel C: shows the satisfied contacts the $top1$ MELD prediction. Green contacts are present in the native structure (true positives) and purple ones are absent (false positive). Panel D: native structure using a blue (N-termini)- white - red (C-termini) coloring scheme.

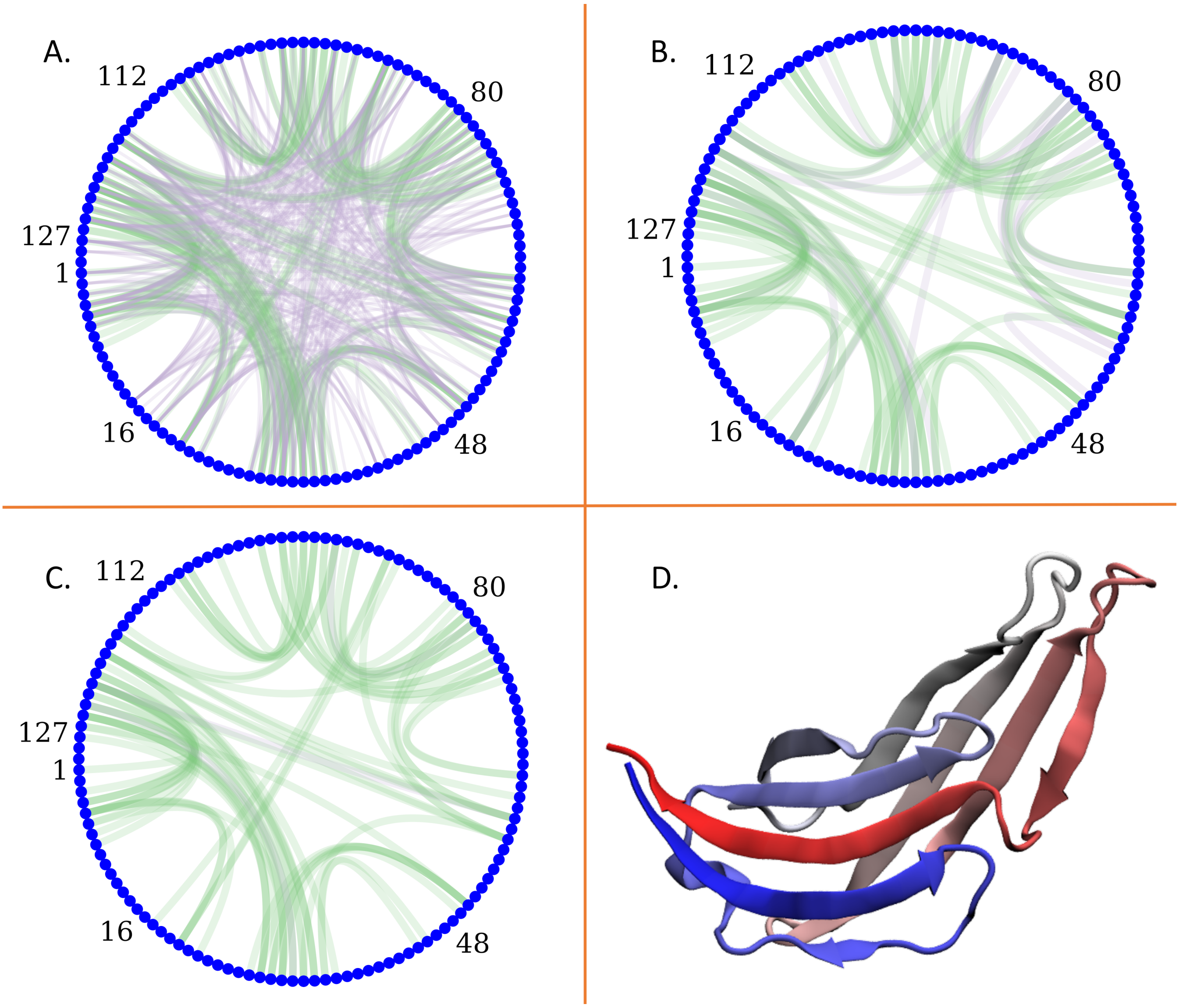

Figure S11. Target: N0981D5. Panels A-C show a graph representation of the protein sequence, with each residue as a blue node. Lines between residues represent contacts. Panel A: all possible contacts in the *4-residue ambiguous dataset*. Green lines represent the correct assignment of the data. Panel B: each line is a true contact in the native structure, shown as either satisfied in the $top1$ cluster (green, true positive) or non-satisfied (purple, false negative). Panel C: shows the satisfied contacts the $top1$ MELD prediction. Green contacts are present in the native structure (true positives) and purple ones are absent (false positive). Panel D: native structure using a blue (N-termini)- white - red (C-termini) coloring scheme.

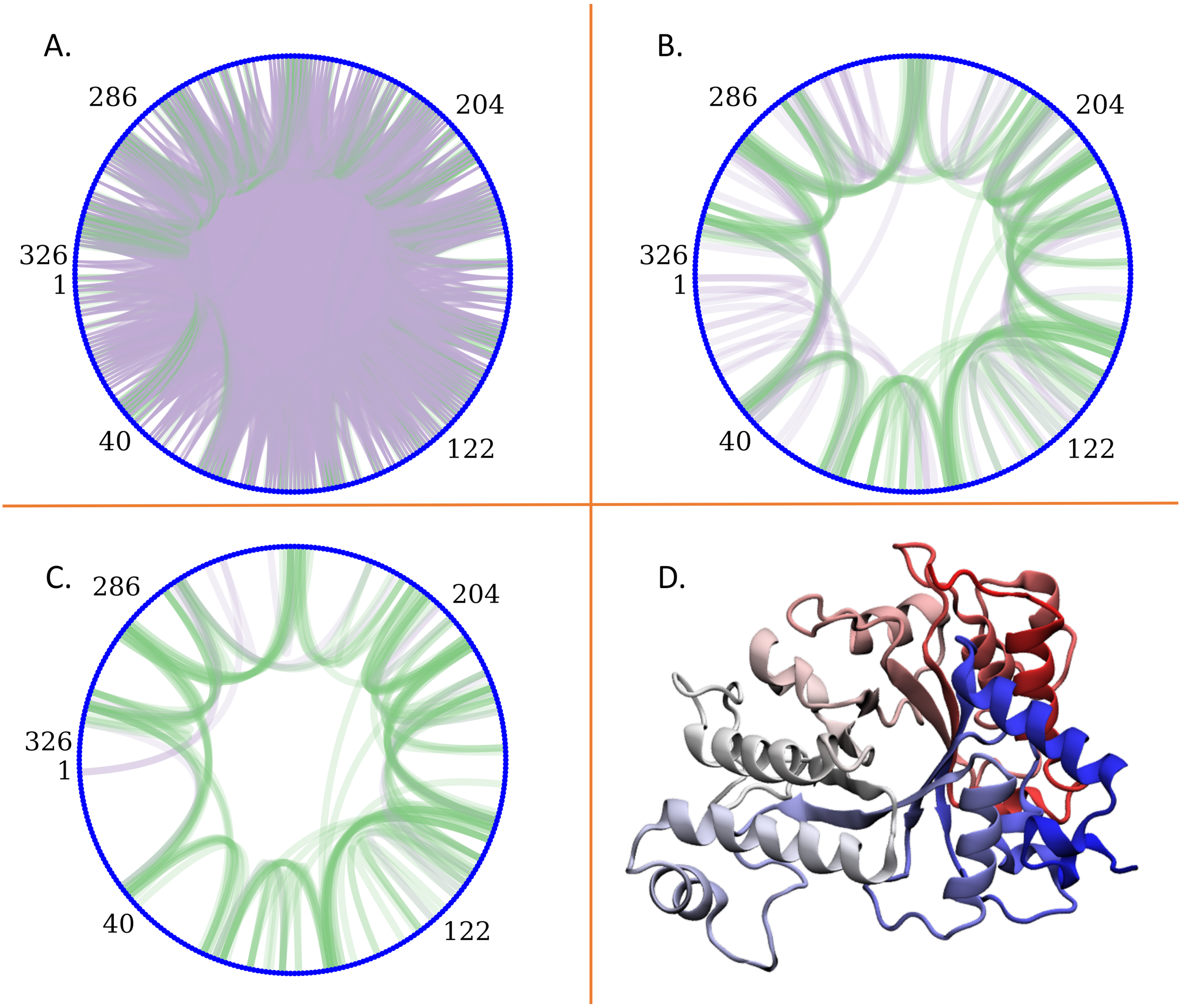

Figure S12. Target: N1005. Panels A-C show a graph representation of the protein sequence, with each residue as a blue node. Lines between residues represent contacts. Panel A: all possible contacts in the *4-residue ambiguous dataset*. Green lines represent the correct assignment of the data. Panel B: each line is a true contact in the native structure, shown as either satisfied in the $top1$ cluster (green, true positive) or non-satisfied (purple, false negative). Panel C: shows the satisfied contacts the $top1$ MELD prediction. Green contacts are present in the native structure (true positives) and purple ones are absent (false positive). Panel D: native structure using a blue (N-termini)- white - red (C-termini) coloring scheme.

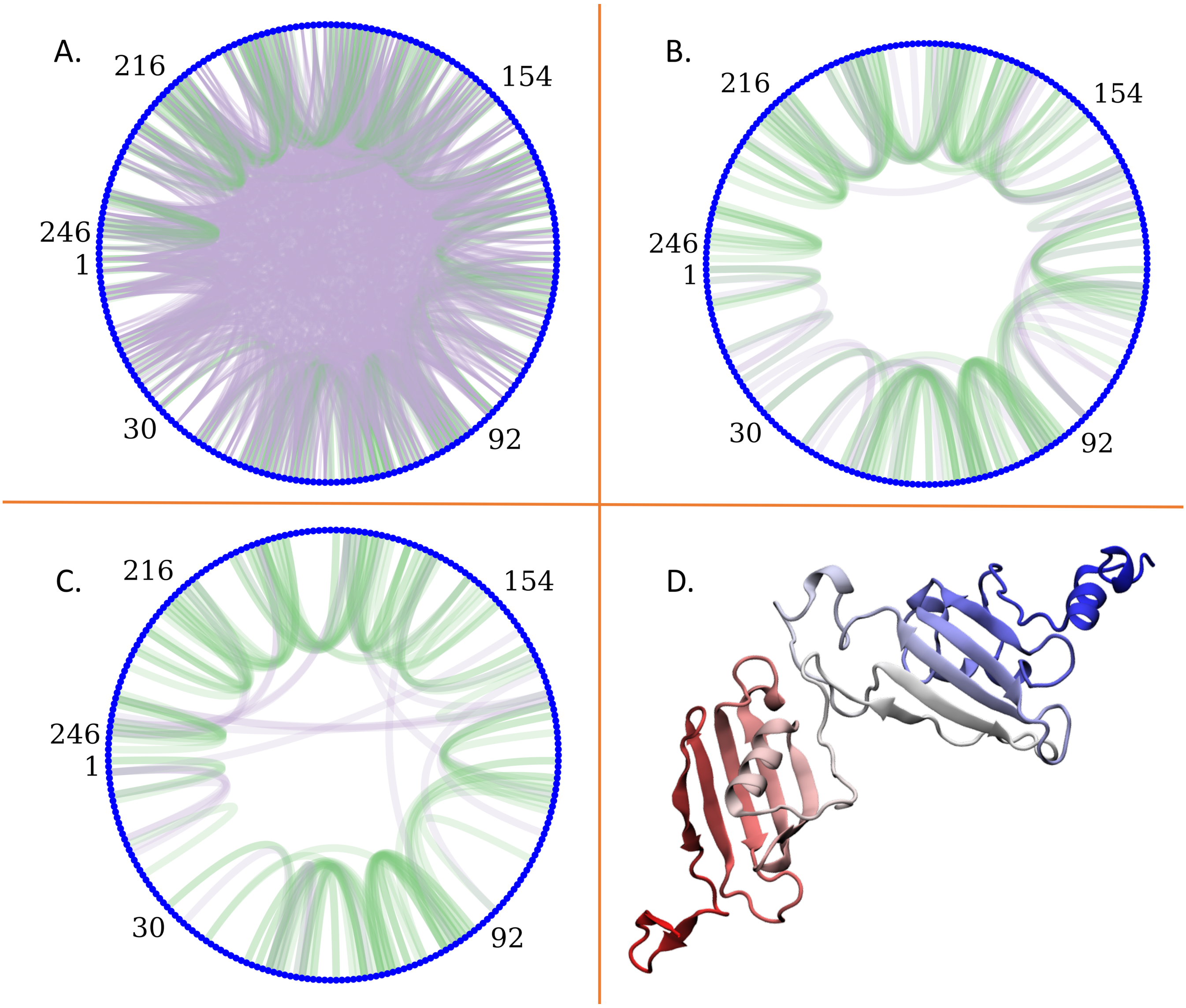

Figure S13. Target: N0989. Panels A-C show a graph representation of the protein sequence, with each residue as a blue node. Lines between residues represent contacts. Panel A: all possible contacts in the *4-residue ambiguous dataset*. Green lines represent the correct assignment of the data. Panel B: each line is a true contact in the native structure, shown as either satisfied in the $top1$ cluster (green, true positive) or non-satisfied (purple, false negative). Panel C: shows the satisfied contacts the $top1$ MELD prediction. Green contacts are present in the native structure (true positives) and purple ones are absent (false positive). Panel D: native structure using a blue (N-termini)- white - red (C-termini) coloring scheme.

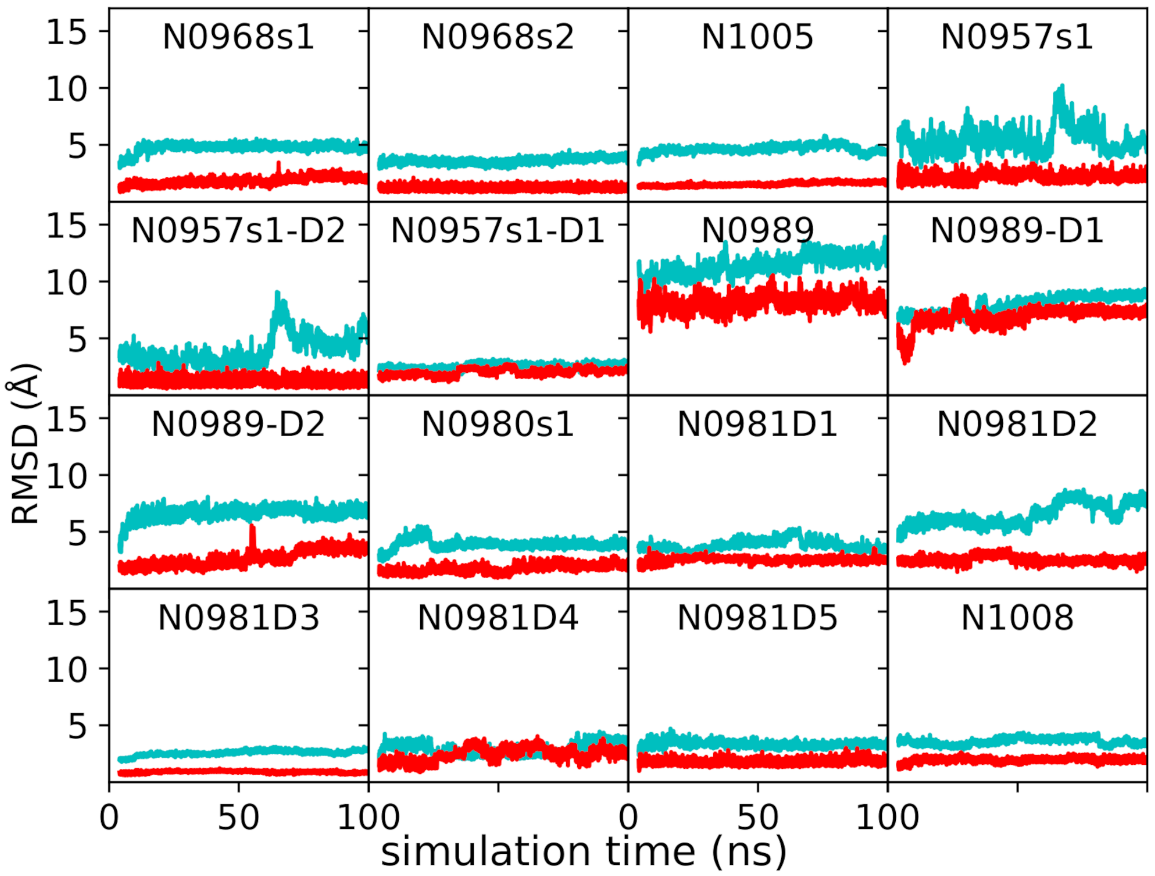

Figure S14. RMSD of each target (and domain) from implicit solvent amber simulations in the presence (red) and absence (cyan) of true NMR data ($4 - residetruedataset$).

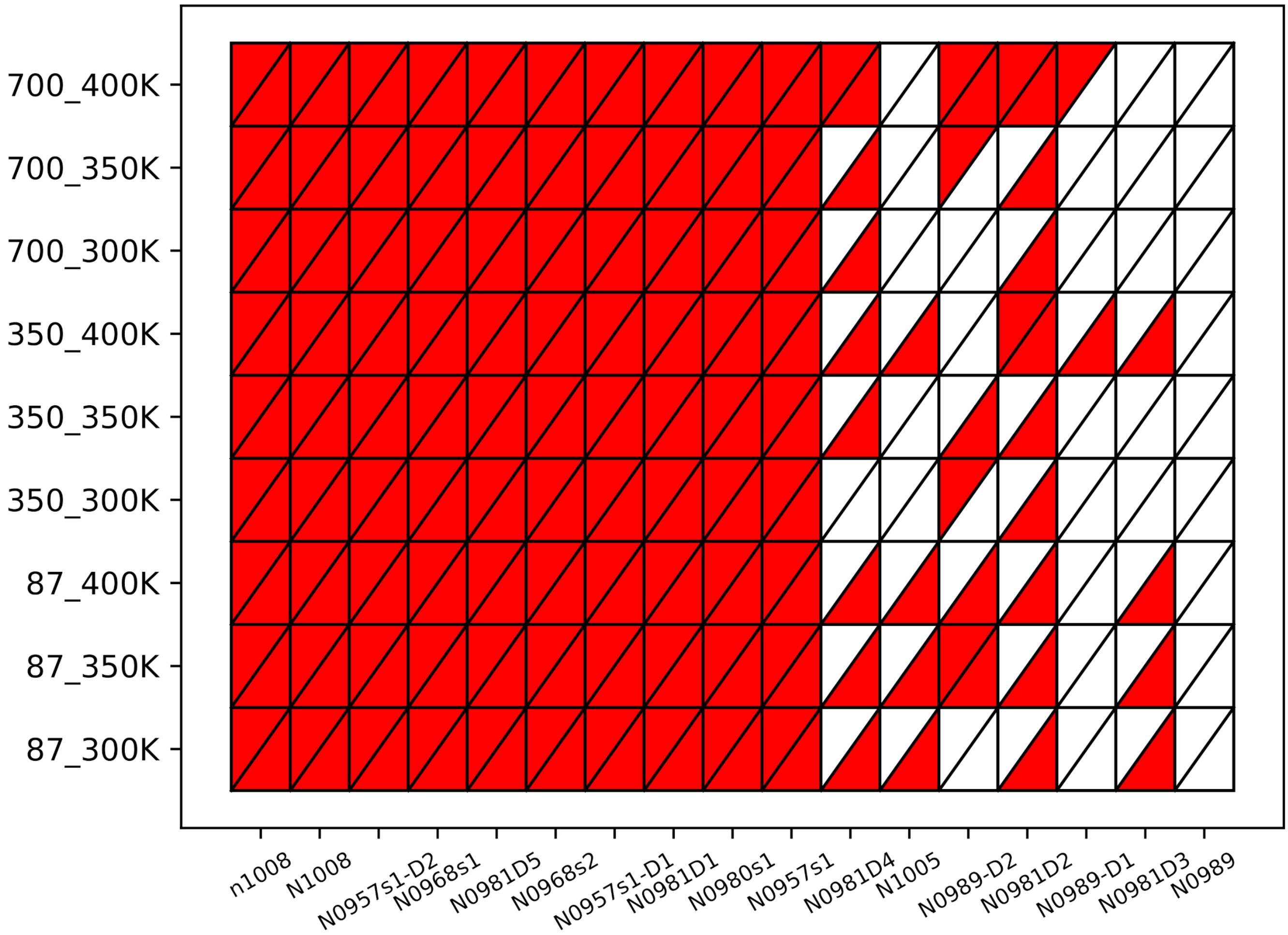

Figure S15. Presence (red) or absence (white) of a native conformation in the whole ensemble (RMSD below 4Å) using either the $4-residueambiguous$ dataset (upper left triangle) and $4-residuetrue$ dataset (lower right triangle). Each line represents a different HT-REMD protocol. Each protocol is described in the y axis using the notation $forceconstant\_tempererature$. Force constants are given in $kJmol^{-1}nm^{-2}$ and temperature in Kelvin.

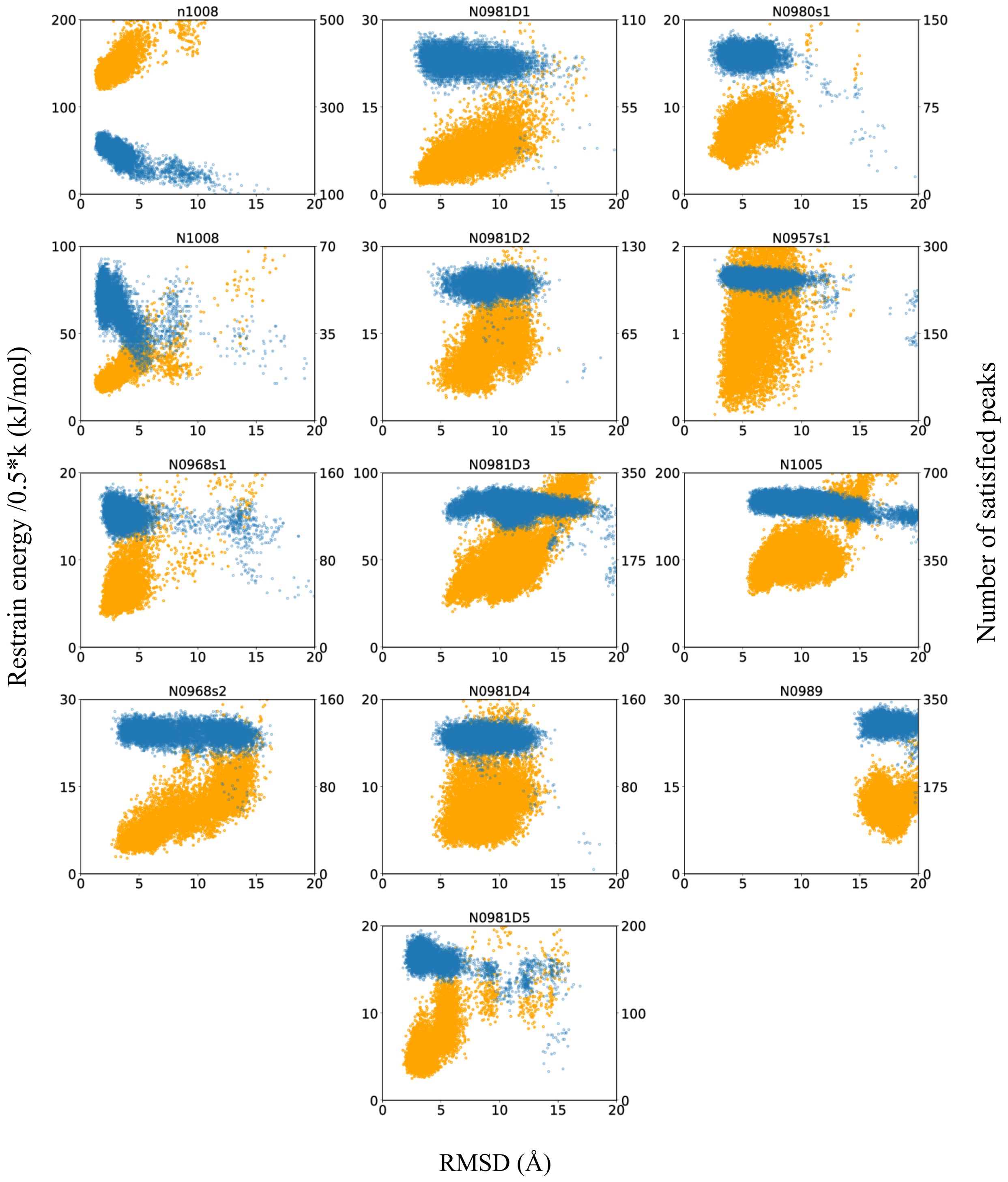

Figure S16. Orange dots represent restraint energy (left y-axis) and blue dots represent the number of satisfied peaks (right y-axis) of each frame for the trial 350 $kJmol^{-1}nm^{-2}$ force constant and 400K temperature protocol.

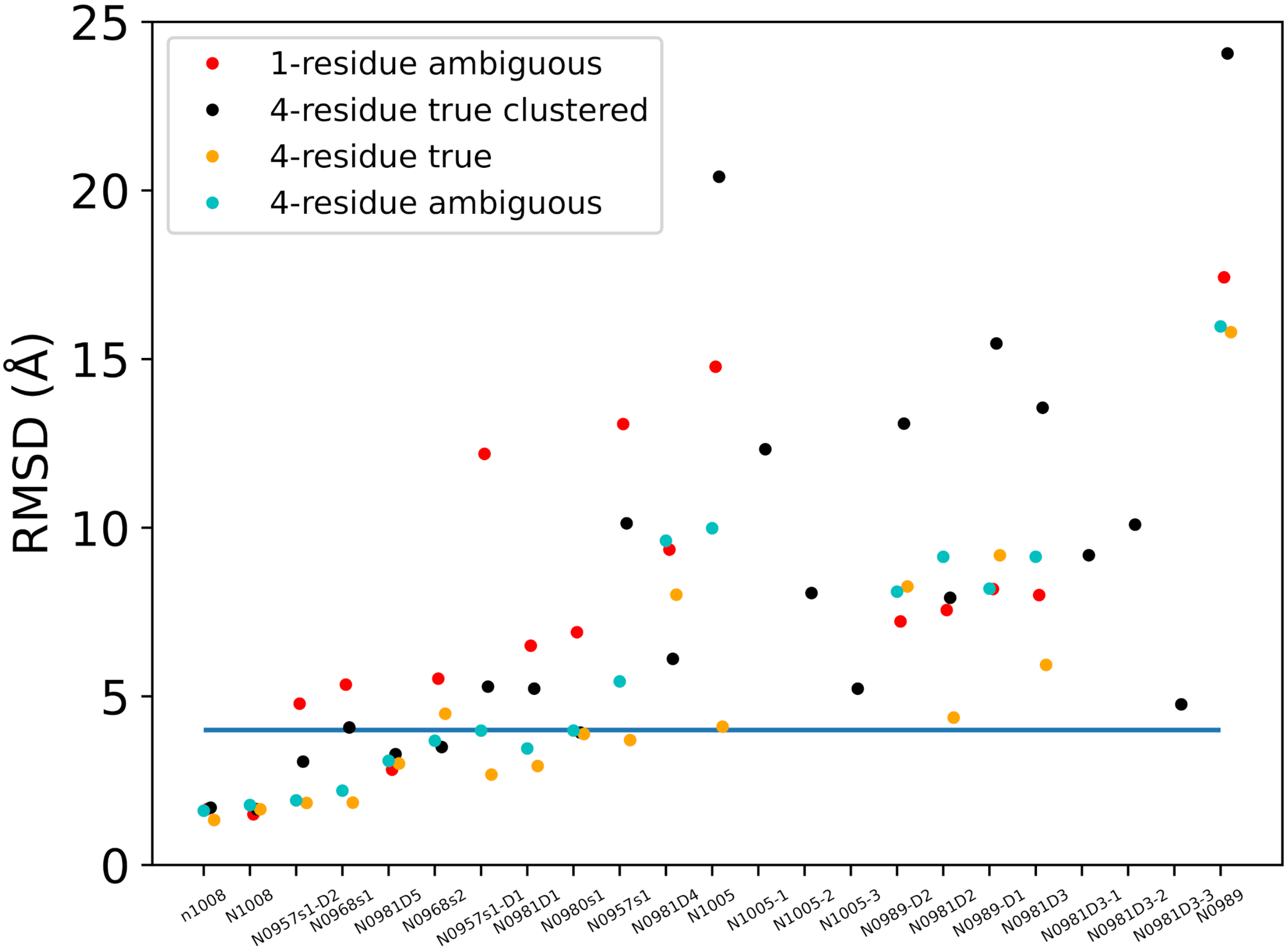

Figure S17. RMSD of all targets using 350 $kJmol^{-1}nm^{-2}$ force constant at 400 K temperature for 4 different NMR datasets. Target N1005-1, N1005-2, and N1005-3 correspond to trial with 25, 50 and 100 contacts and target N0981D3-1, N0981D3-2, and N0981D3-3 correspond to trial with 20,30 and 50 contacts from *4-residue true clustered dataset*.

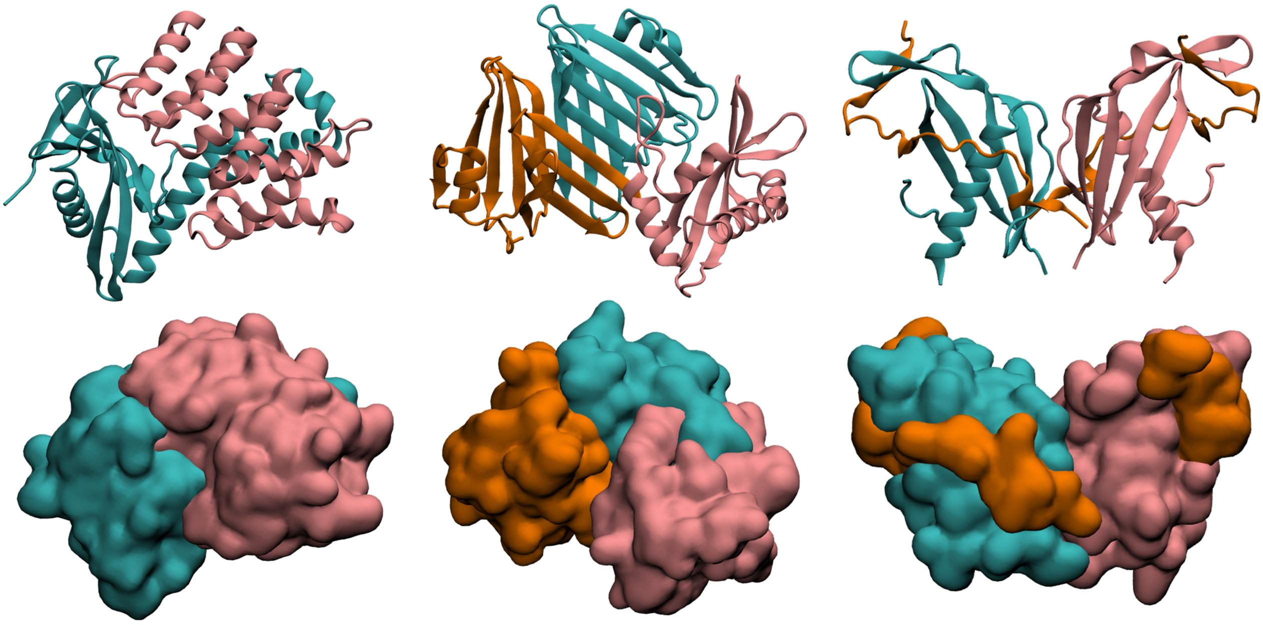

N0957 N0968 N0980

Figure S18. Multimer complexes of three targets in new cartoon (top row) and surface (bottom row) representation. For N0957, cyan represents N0957s1 and pink represents its interacting partner, N0957s2. For N0968, cyan represent the N0968s2 which stabilizes the terminal strand by interacting with another unit of N0968s2 shown in orange. It also interacts with N0968s1 (pink) which helps it to stabilize a loop region. For N0980, two units of N0980s1 interact with each other, shown in cyan and pink, and each interacts with a short protein chain (orange) stabilizing one of the β-sheets.

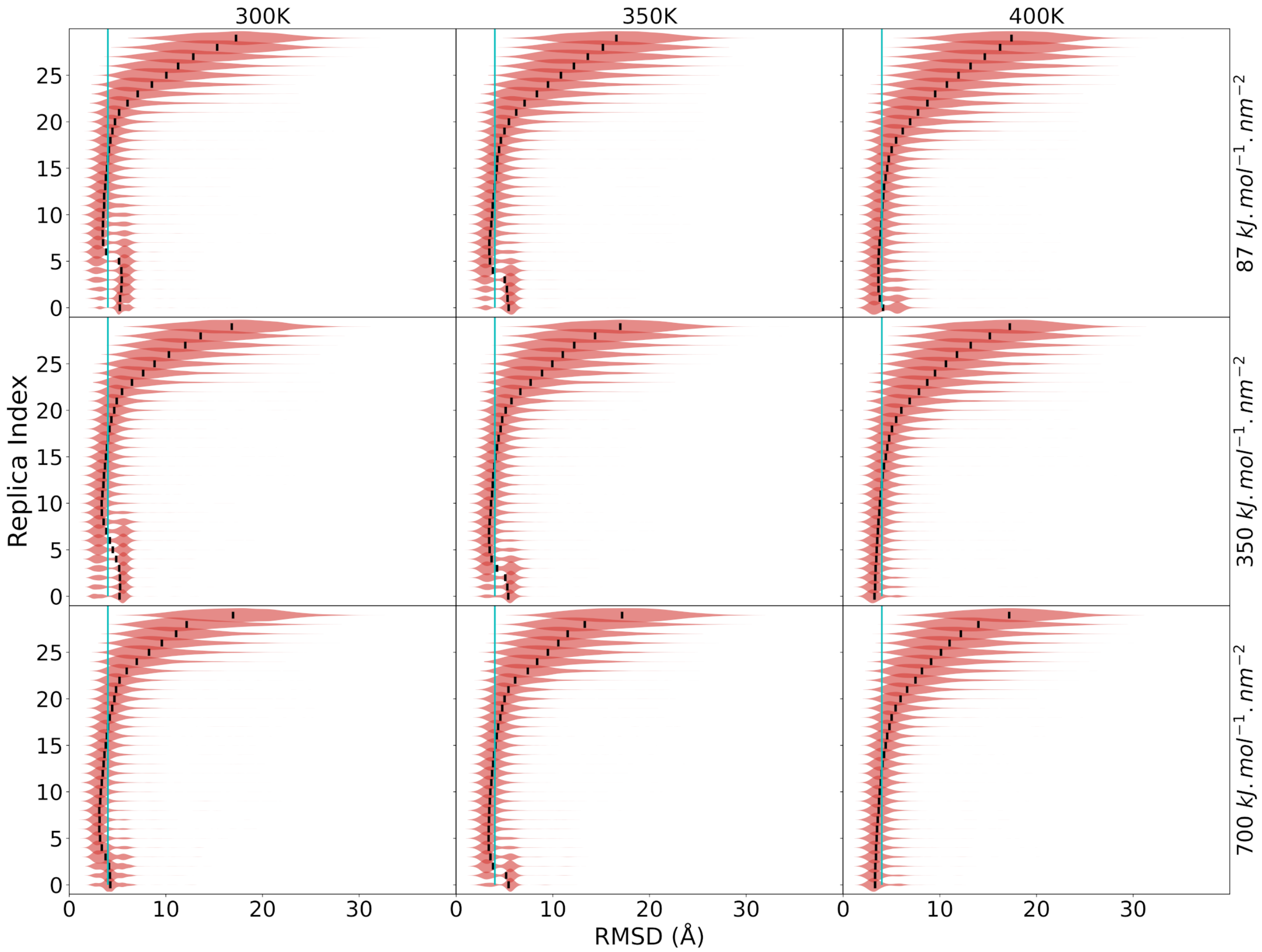

Figure S19. Target: N0957s1-D1 RMSD distribution (red) of each replica for all 9 protocols using the $4 - residueambiguous$ NMR dataset. The black line in each replica shows the median and the cyan line represents 4Å.

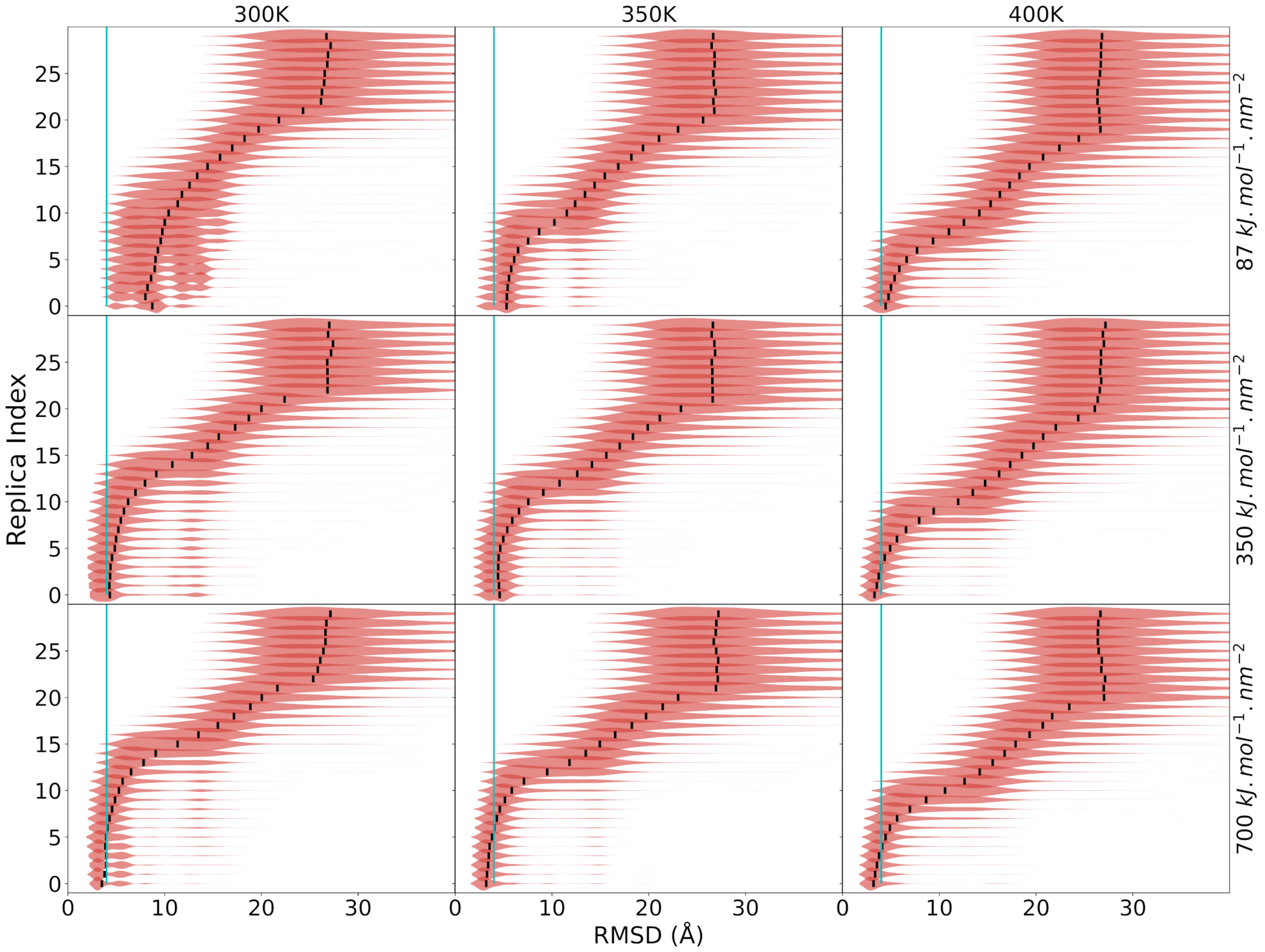

Figure S20. Target: N0968s1. RMSD distribution (red) of each replica for all 9 protocols using the $4 - residueambiguous$ NMR dataset. The black line in each replica shows the median and the cyan line represents 4Å.

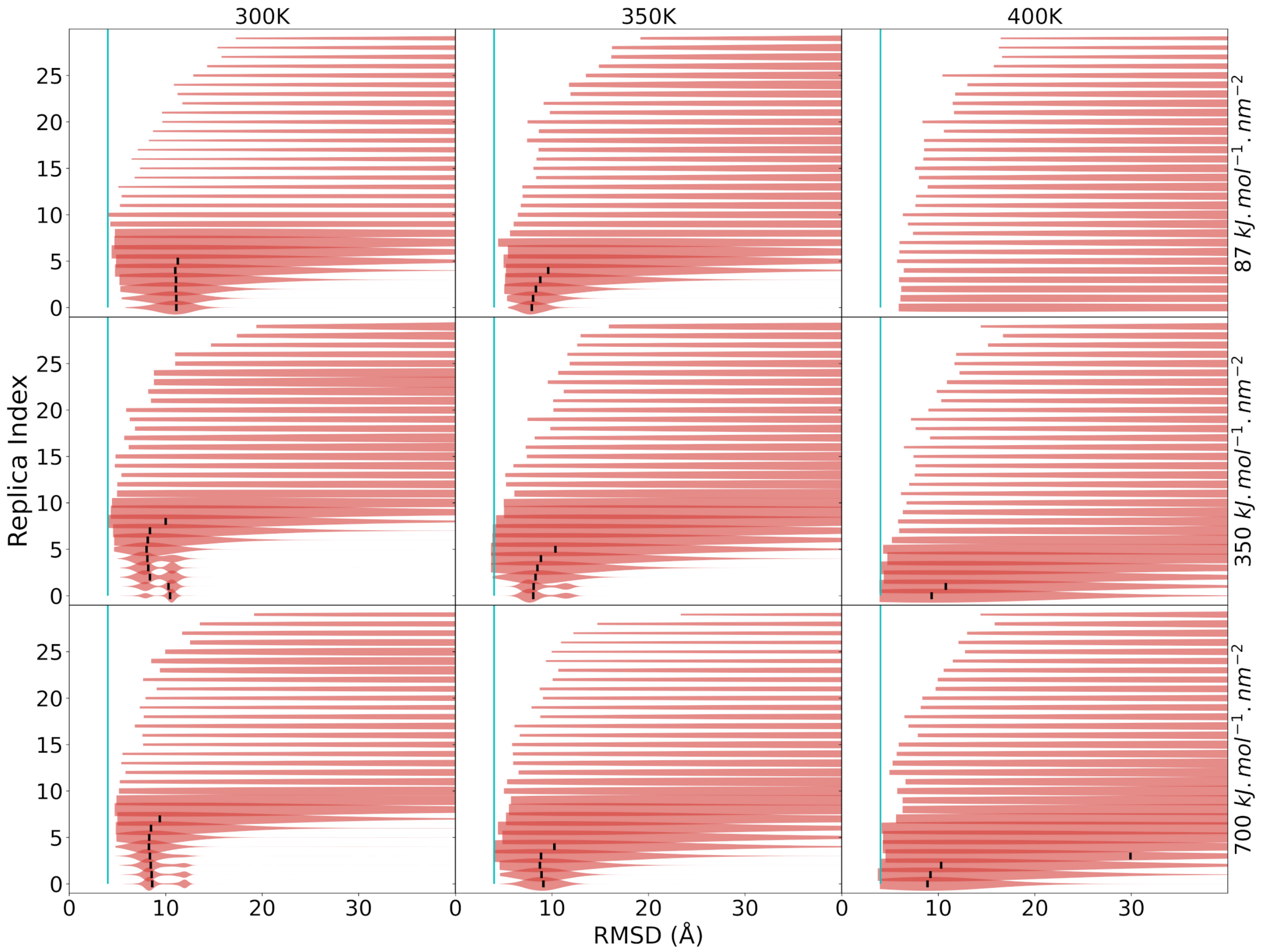

Figure S21. Target: N0981D2. RMSD distribution (red) of each replica for all 9 protocols using the $4 - residueambiguous$ NMR dataset. The black line in each replica shows the median and the cyan line represents 4Å.

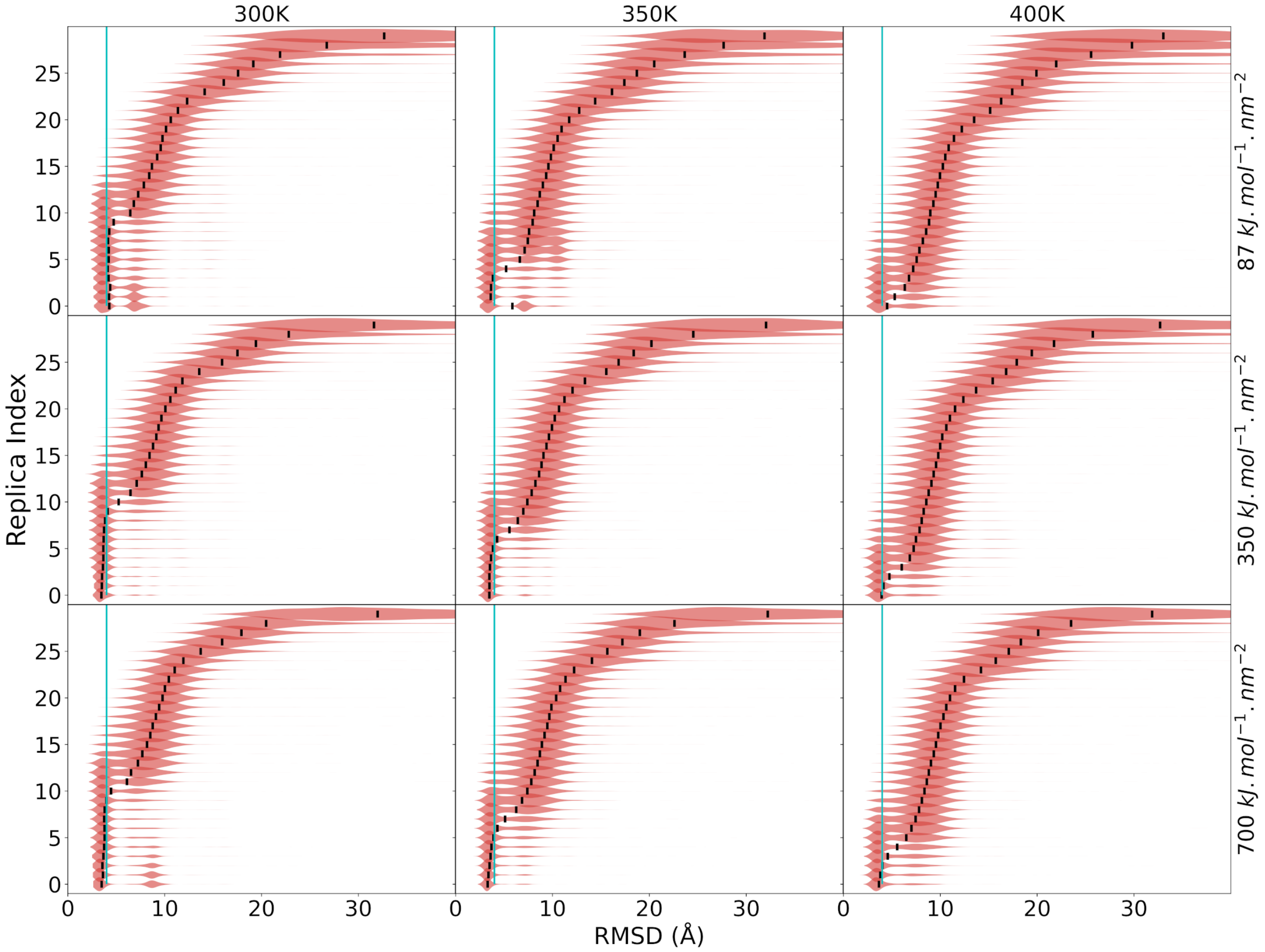

Figure S22. Target: N0957s1-D2. RMSD distribution (red) of each replica for all 9 protocols using the $4 - residueambiguous$ NMR dataset. The black line in each replica shows the median and the cyan line represents 4Å.

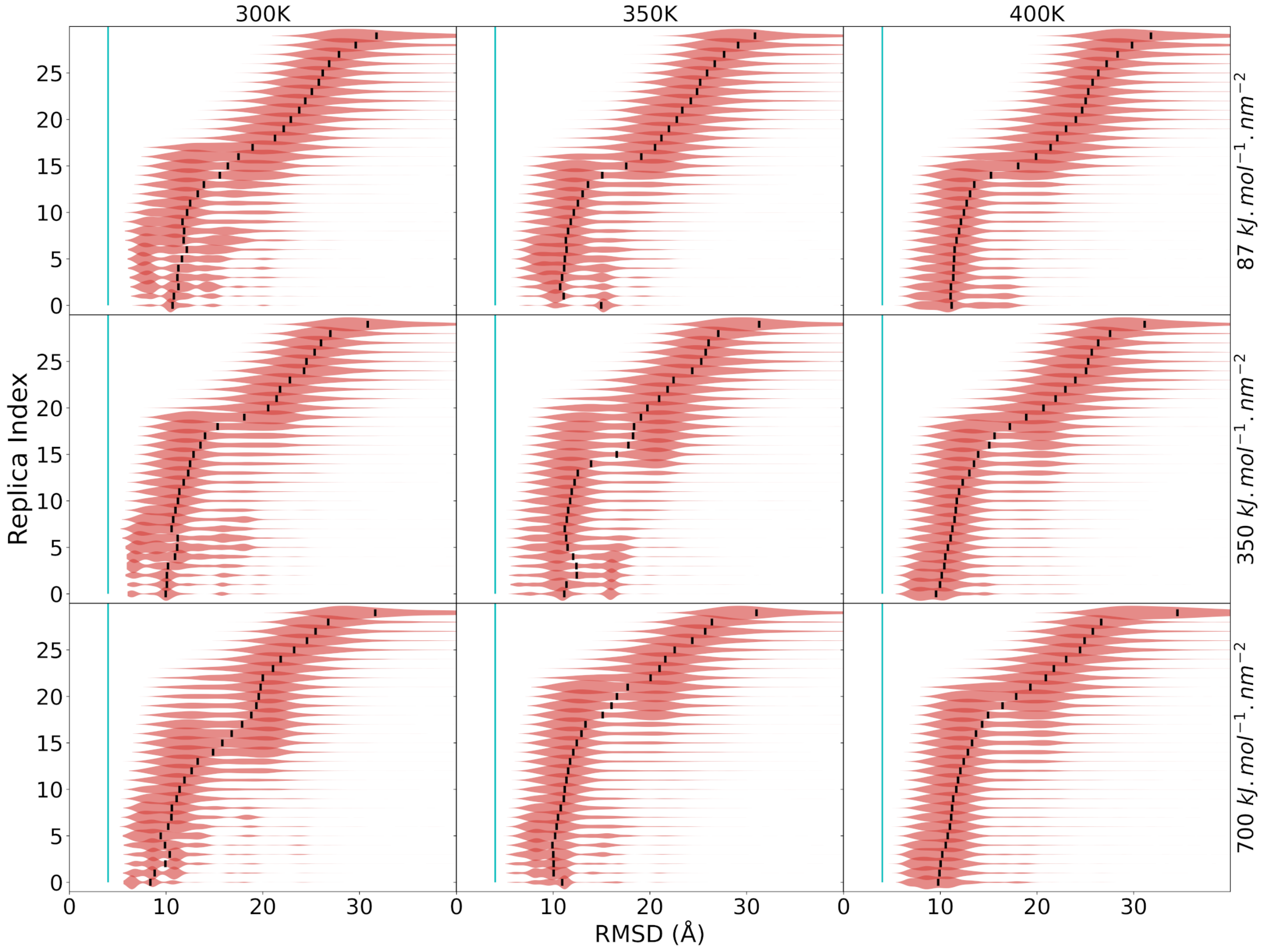

Figure S23. Target: N1005. RMSD distribution (red) of each replica for all 9 protocols using the $4-residueambiguous$ NMR dataset. The black line in each replica shows the median and the cyan line represents 4Å.

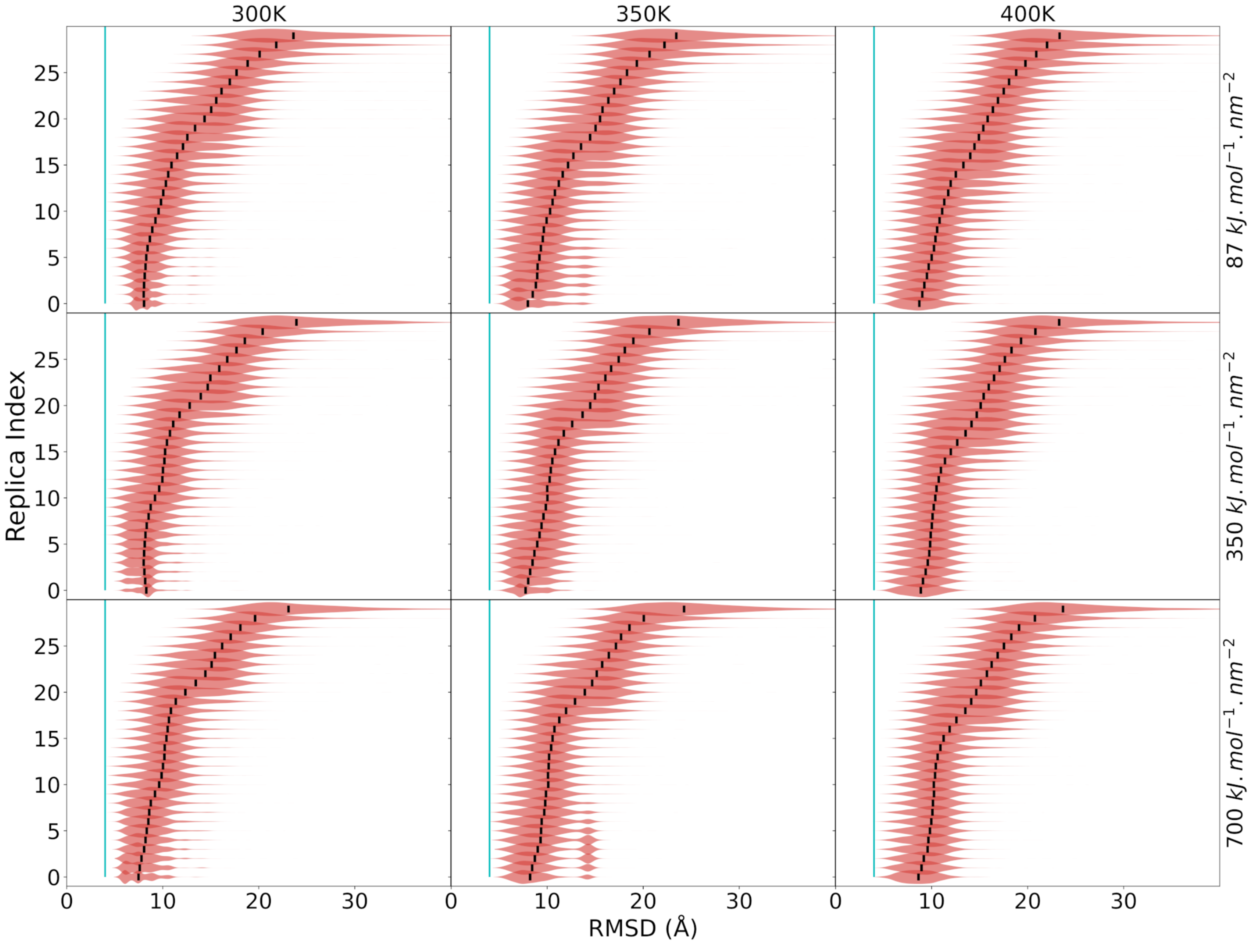

Figure S24. Target: N0981D4. RMSD distribution (red) of each replica for all 9 protocols using the $4 - residueambiguous$ NMR dataset. The black line in each replica shows the median and the cyan line represents 4Å.

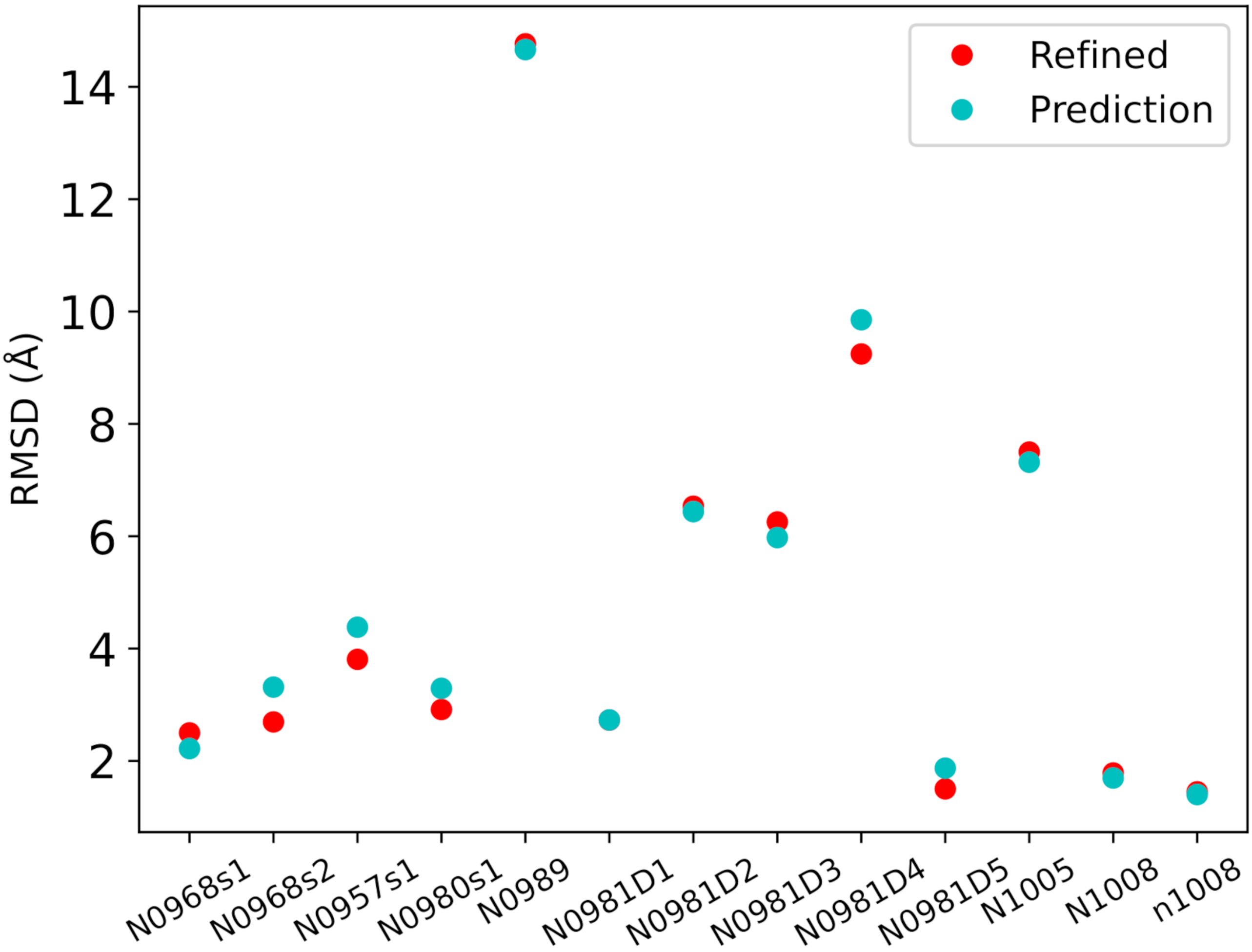

Figure S25. RMSD of the MELD prediction (cyan) and after explicit solvent refinement (red). Residues with no secondary structure are excluded from the RMSD analysis.

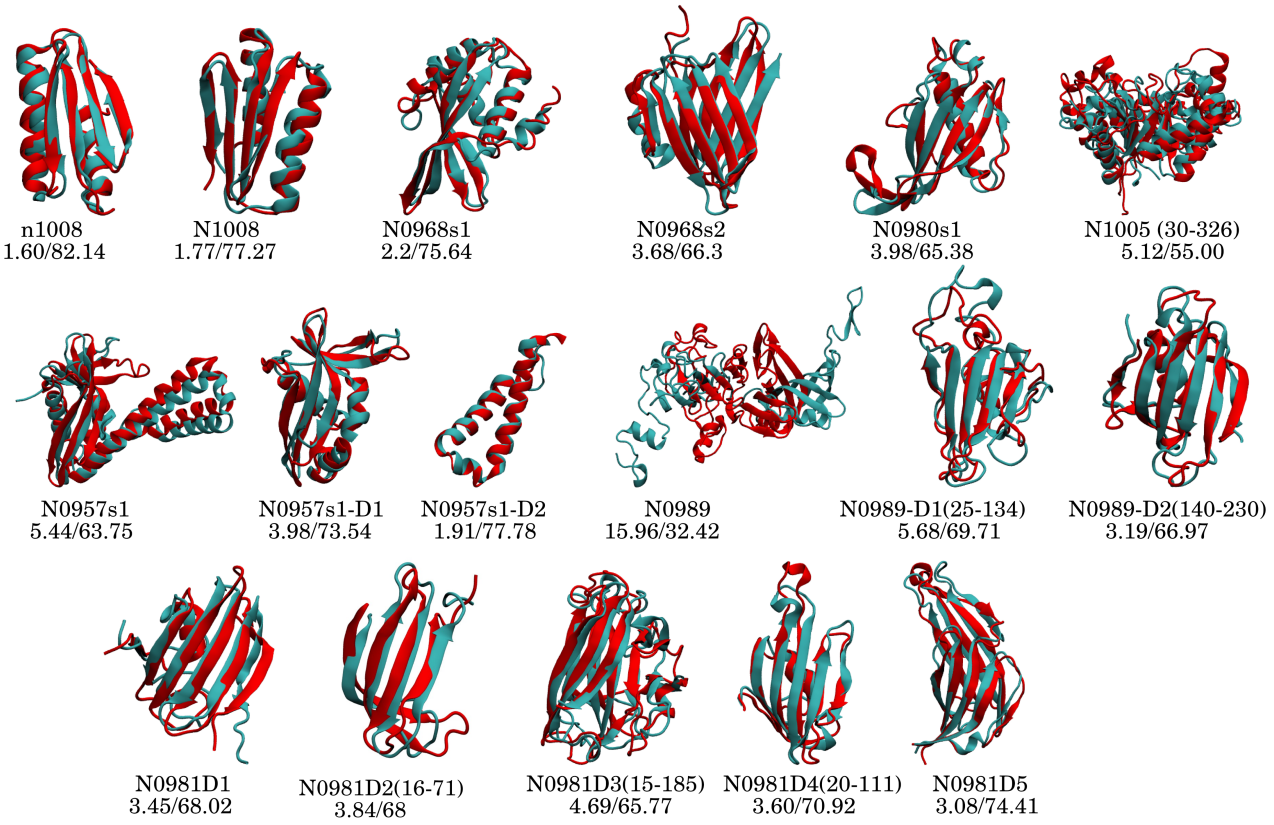

Figure S26. Superposition of native (cyan) and prediction (red) for all targets and domains ignoring unstructured termini. The first numbers correspond the Cα RMSD from the native and the second number represent the GDT-TS score. When present, the numbers in the parenthesis refer to the residue range we used in the analysis. Otherwise, the whole sequence was used.
